# Whole‐genome resequencing reveals the pleistocene temporal dynamics of *Branchiostoma belcheri* and *Branchiostoma floridae* populations

**DOI:** 10.1002/ece3.6527

**Published:** 2020-07-20

**Authors:** Changwei Bi, Na Lu, Zhen Huang, Junyuan Chen, Chunpeng He, Zuhong Lu

**Affiliations:** ^1^ State Key Laboratory of Bioelectronics School of Biological Science and Medical Engineering Southeast University Nanjing China; ^2^ The Public Service Platform for Industrialization Development Technology of Marine Biological Medicine and Product of State Oceanic Administration College of Life Sciences Fujian Normal University Fuzhou China; ^3^ Key Laboratory of Special Marine Bio‐resources Sustainable Utilization of Fujian Province Fuzhou China; ^4^ Nanjing Institute of Paleontology and Geology Chinese Academy of Sciences Nanjing China

**Keywords:** *Branchiostoma belcheri*, *Branchiostoma floridae*, climatic fluctuations, demographic history, effective population size, genomic variations

## Abstract

Global climatic fluctuations governed the ancestral demographic histories of species and contributed to place the current population status into a more extensive ecological and evolutionary context. Genetic variations will leave unambiguous signatures in the patterns of intraspecific genetic variation in extant species since the genome of each individual is an imperfect mosaic of the ancestral genomes. Here, we report the genome sequences of 20 *Branchiostoma* individuals by whole‐genome resequencing strategy. We detected over 140 million genomic variations for each *Branchiostoma* individual. In particular, we applied the pairwise sequentially Markovian coalescent (PSMC) method to estimate the trajectories of changes in the effective population size (*N*_e_) of *Branchiostoma* population during the Pleistocene. We evaluated the threshold of sequencing depth for proper inference of demographic histories using PSMC was ≥25×. The PSMC results highlight the role of historical global climatic fluctuations in the long‐term population dynamics of *Branchiostoma*. The inferred ancestral *N*_e_ of the *Branchiostoma belcheri* populations from Zhanjiang and Xiamen (China) seawaters was different in amplitude before the first (mutation rate = 3 × 10^−9^) or third glaciation (mutation rate = 9 × 10^−9^) of the Pleistocene, indicating that the two populations most probably started to evolve in isolation in their respective seas after the first or third glaciation of the Pleistocene. A pronounced population bottleneck coinciding with the last glacial maximum was observed in all *Branchiostoma* individuals, followed by a population expansion occurred during the late Pleistocene. Species that have experienced long‐term declines may be especially vulnerable to recent anthropogenic activities. Recently, the industrial pollution and the exploitation of sea sand have destroyed the harmonious living environment of amphioxus species. In the future, we need to protect the habitat of *Branchiostoma* and make full use of these detected genetic variations to facilitate the functional study of *Branchiostoma* for adaptation to local environments.

## Graphical abstract

This study reported the genome sequences of twenty *Branchiostoma* individuals by whole‐genome resequencing strategy and applied the pairwise sequentially Markovian coalescent (PSMC) method to estimate the trajectories of changes in the effective population size (*N*_e_) of *Branchiostoma* population during the Pleistocene.

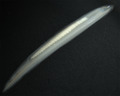

## 1 INTRODUCTION

The Quaternary Period followed the Neogene Period and spans from approximately 2.58 million years ago (Mya) to the present. It can be divided into two epochs: the Pleistocene (2.58 Mya to 11.7 kya) and the Holocene. During the Quaternary Period, global climatic fluctuations influenced the demography and distribution of biodiversity on Earth (Farrand, 1984; Nadachowska‐Brzyska, Li, Smeds, Zhang, & Ellegren, 2015). In glacial periods, many species suffered from extreme contraction or disappearance of their survival habitats (Hewitt, 2000; Hewitt, 2004; Holm & Svenning, 2014). As a consequence, these species went extinct or had to migrate to new areas where they survived in glacial refugia and adapted to the new conditions (Arenas, Ray, Currat, & Excoffier, 2012). In interglacial periods, large areas of suitable habitats became available again giving rise to the re‐expansion of many species. The frequent species migration in glacial and interglacial periods likely led to several different genetic consequences (Arenas et al., 2012; Hewitt, 2004). For example, many previously large populations may have undergone a decrease in genetic diversity due to genetic bottlenecks in glacial refugia or the “founder effect” during postglacial expansions (Provine, 2004; Templeton, 1980). Additionally, the geographical separation of populations in glacial refugia likely accelerated the population differentiation and, in some cases, led to allopatric speciation (Hewitt, 2004). Some newly formed species came into contact with other species during interglacial expansions and sometimes formed stable hybrid regions (Hewitt, 2010).

Understanding ancestral demographic histories and climatic changes allow us to place the current population status into a more extensive ecological and evolutionary context. Many studies have demonstrated that genetic variation in abundance leaves unambiguous signatures in the patterns of intraspecific genetic variation in extant species since the genome of each individual is an imperfect mosaic of the ancestral genomes (Hewitt, 2004; Li & Durbin, 2011; Sheehan, Harris, & Song, 2013). As a consequence, we can infer the historical effective population sizes (*N*_e_) using a whole‐genome resequencing strategy. In the first decade of the 21st century, many approaches were developed to analyze the demographic history of species using genetic data (Beaumont & Zhang, 2002; Csillery, Blum, Gaggiotti, & Francois, 2010; Emerson, Paradis, & Thebaud, 2001; Jody & Machado, 2003). However, these methods are restricted to special molecular markers, *that is*, a limited number of genomic loci instead of whole‐genome data (Drummond, Rambaut, Shapiro, & Pybus, 2005; Heled & Drummond, 2008; Jody & Rasmus, 2004; Nikolic & Chevalet, 2014; Pybus, Rambaut, & Harvey, 2000). To accurately estimate the demographic history and timing of speciation, it is important to sample large numbers of genomic loci throughout the whole genome (Arbogast, Edwards, Wakeley, Beerli, & Slowinski, 2002; Ballard & Whitlock, 2004; Edwards & Beerli, 2010). In 2011, the pairwise sequentially Markovian coalescent (PSMC) model was developed to estimate the changes in effective population size through time using whole‐genome data (Li & Durbin, 2011). The PSMC model uses a hidden Markov model to detect the historical recombination events across a single diploid genome. The hidden state of a given position corresponds to the coalescence time of the two lineages at that position, while the observed state corresponds to the observed genotype (homozygous/heterozygous) at that position. Since species evolve with the genome sequence, the coalescence time may change as a result of recombination. Moreover, the spatial distribution of homozygous and heterozygous loci will provide information on the distribution of coalescence times, which depends on the past population sizes. Although the PSMC model can produce a reasonably accurate estimation of the effective population sizes, estimating population sizes in the most recent past (<20 kya) or more anciently than 3 Mya ago is hampered, because only few recombination events are left in the present sequence. The lack of sufficient recombination events in the most recent past reduces the power of the PSMC model to satisfactorily infer the species demographic history below 20  kya (Li & Durbin, 2011). The PSMC model has been widely used in many vertebrate genome sequencing projects. For example, in the Tibetan macaque, giant pandas, and crested ibis, PSMC was used to investigate the demographic histories of these endangered species (Fan et al., 2014; Li et al., 2014; Nadachowska‐Brzyska et al., 2015; Zhao et al., 2013); in brown rat (*Rattus norvegicus*), PSMC was used to detect the ancestral population declines or expansions to assist in the interpretation of population genomic statistics (Deinum et al., 2015); in pied flycatcher, collared flycatcher, and dogs, PSMC was used to complement other demographic history inferences (Freedman et al., 2014; Nadachowska‐Brzyska et al., 2013). However, in spite of its wide usage in vertebrates, PSMC approach has not been applied in cephalochordates to infer demographic history.

**FIGURE 1:**
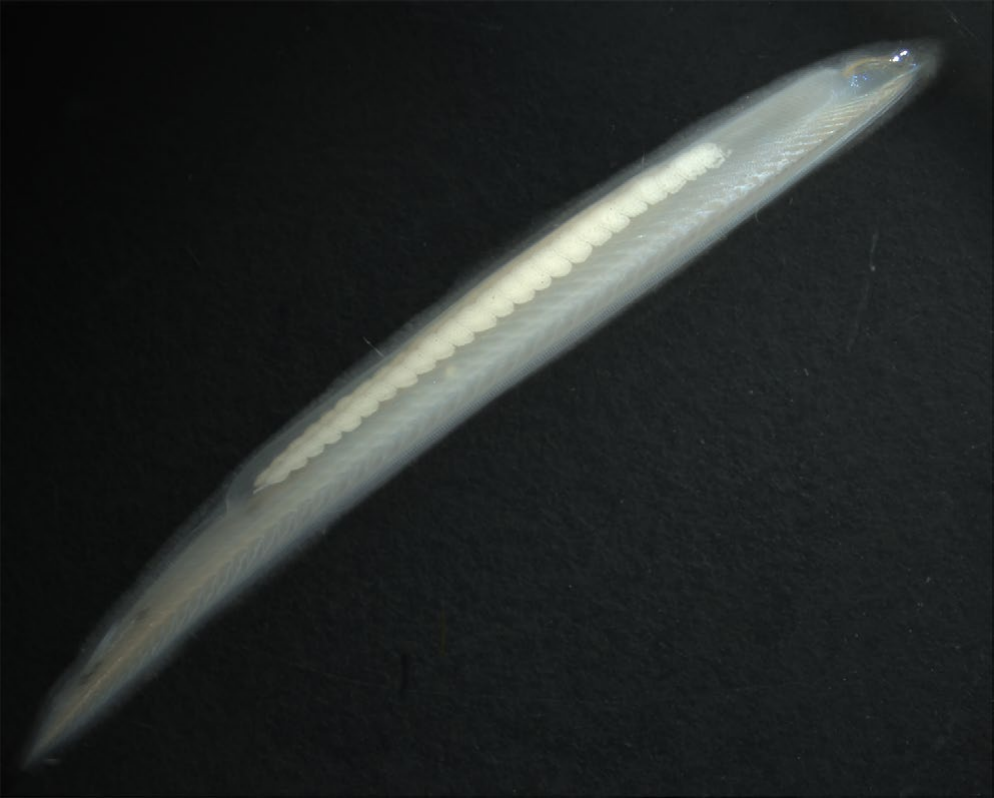
The amphioxus of *Branchiostoma belcheri* from Xiamen, China. The picture was taken in the laboratory

Cephalochordates occupy a key evolutionary position close to the ancestral chordates. They have been placed as the most basal chordate group through molecular phylogenetic analyses (Alexandre & Pierre, 2006; Bertrand & Escriva, 2011; Blair & Hedges, 2005; Frédéric, Henner, Daniel, & Hervé, 2006; Putnam et al., 2008). Cephalochordate, commonly called amphioxus or lancelet, has long been regarded as the phylogenetic model organism for understanding the evolution of vertebrate development (Holland et al., 2008; Huang et al., 2014; Marletaz et al., 2018; Putnam et al., 2008; Yuan, Zhang, Guo, Wang, & Shen, 2015). Lancelets, small vaguely fish‐shaped creatures (Figure 1), are mainly distributed in shallow subtidal sand flats in temperate, subtropical, and tropical seas around the world (Carvalho, Lahaye, & Schubert, 2017). They live in sandy bottoms whose granulometry depends on the species and the site, and they are usually found half‐buried in sand (Escriva, 2018). Unlike the much‐reduced metamorphosis of the urochordates, amphioxus species seem to have been maintained in a morphological stasis for several hundred million years. This can be evidenced by the fossil record, which has brought to light a number of Cambrian soft‐body fossils that closely resemble the extant amphioxus (Garcia‐Fernandez & Benito‐Gutierrez, 2009). Therefore, studies on amphioxus species are useful for investigating the conserved patterning mechanisms for chordates (Schubert, Escriva, Xavier‐Neto, & Laudet, 2006). Currently, over 30 amphioxus species have been recorded worldwide and can be subdivided into three genera: *Branchiostoma*, *Epigonichthys*, and *Asymmetron*. The majority of amphioxus species are in the genus *Branchiostoma*. In some areas, the amphioxus populations were previously quite large. Before the 1950s, they were commercially harvested from fisheries in Xiamen, China. However, during the industrial era, pollution and exploitation of sea sand destroyed the harmonious living environment for the largest populations of amphioxus, and thus, amphioxus has been listed in the registry of “State Second‐Class Protected Animals” in China and “Endangered Animals of Japanese Marine and Fresh Water Organisms” in Japan (Hantao, Yuanyuan, Chen, Fan, & Yuwu, 2005; Kubokawa, Azuma, & Tomiyama, 1998).

The recently released whole‐genome sequences of *Branchiostoma belcheri* (*B. belcheri*) and *Branchiostoma floridae* (*B. floridae*) provide valuable references for the in‐depth analyses of the ancestral population dynamics of *Branchiostoma*. In this study, we investigated five Zhanjiang *B. belcheri* individuals, five Xiamen *B. belcheri* individuals, and ten *B. floridae* individuals to infer the changes in the amphioxus population sizes during the Pleistocene using the PSMC model. We first detected and annotated the genome‐wide genetic variations of each individual, then estimated the best parameters for the PSMC model, and then inferred the influence of ancestral climatic fluctuations on the population sizes of *B. belcheri* and *B. floridae*. We also further analyzed the different patterns in the ancestral *N*_e_ of *B. belcheri* and *B. floridae* populations to gain insight into the adaptation to different climates.

## 2 MATERIAL AND METHODS

### 2.1 Ethics statement

All sample collecting and processing were performed according to the local laws governing the welfare of invertebrate animals and were approved by the Southeast University (Nanjing, China) ethical committee.

### 2.2 Samples preparation and sequencing

Five wild *B. belcheri* (BB_ZJ1, BB_ZJ2, BB_XM3, BB_XM4, and BB_XM5) collected from Zhanjiang and Xiamen seawater, China, and five laboratory‐raised *B. floridae* (BF_CN1, BF_CN2, BF_CN3, BF_CN4, and BF_CN5) from Fuzhou, China, were used for whole‐genome resequencing. The laboratory‐raised *B. floridae* individual was an offspring of recently captured *B. floridae* individuals from Tampa Bay, FL, USA. Genomic DNA was extracted from muscular tissues using the QIAamp^®^ DNA Mini kit (Qiagen) following the manufacturer's protocol. Two paired‐end libraries (BB_ZJ1 and BB_ZJ2) with insert sizes of approximately 700 bp and eight (BB_XM3‐5 and BF_CN1‐5) with an insert size of approximately 350 bp were generated, respectively. For each sample, over 100 paired‐end reads were generated using the Illumina HiSeq 2000 platform (Illumina) by the Novogene Bioinformatics Institute, Beijing, China. The other short read sequences of three *B. belcheri* individuals from Zhanjiang (BB_ZJ3, BB_ZJ4, and BB_ZJ5), two *B. belcheri* from Xiamen, China (BB_XM1 and BB_XM2) and five *B. floridae* from Tampa Bay, USA (BF_TP1, BF_TP2, BF_TP3, BF_TP4, and BF_TP5) used in this study were downloaded from the National Center for Biotechnology Information (NCBI). Among the five *B. floridae* individuals from Tampa Bay, BF_TP3, BF_TP4, and BF_TP5 are progenies of BF_TP1 and BF_TP2. For downstream analyses, we also utilized sequence information from the *B. belcheri* v.18h27 (NCBI Assembly: GCF_001625305.1) and *B. floridae* v.2.0 (NCBI Assembly: GCF_000003815.1) reference genomes.

### 2.3 Sequence data preprocessing and mapping

In order to filter the low‐quality paired reads, the sequencing raw reads were first filtered with the following criteria: (a) Reads with unidentified nucleotides (N) exceeding 10% were removed, and (b) reads with more than 50% bases having a phred score ≤ 5 were removed. After quality control, all of the clean paired‐end reads of *B. belcheri* and *B. floridae* were mapped to *B. belcheri* v.18h27 and *B. floridae* v.2.0 reference genomes, respectively, using BWA‐MEM (v0.7.15) with the following parameters: ‐M, ‐k 19 (Li, 2013). Then, we used sambamba to filter secondary and supplementary alignments from the above‐generated SAM files with the following parameters: ‐F "not (secondary_alignment or supplementary)" ‐p ‐l 9 (Tarasov, Vilella, Cuppen, Nijman, & Prins, 2015). The filtered SAM format files were sorted and converted into BAM format files using SAMtools v1.3.1 (Li et al., 2009), and then imported to the GATK MarkDuplicates module (v.4.0.2.1) to mark duplicate reads (McKenna et al., 2010). Finally, we used the Qualimap bamqc tool to estimate the mapped reads and their genomic properties (Okonechnikov, Conesa, & Garcia‐Alcalde, 2016).

### 2.4 Variant detection, filtration, and annotation

In order to improve the quality of variants reported, we performed variant calling following the GATK's best practice pipeline, including Recalibrate Base Quality Scores (BQSR), Call Variants Per‐Sample, and Filter Variants. Since there was no known dbSNP dataset of *Branchiostoima* for BQSR, we generated a SNP dataset as the known dbSNP dataset with the following steps: (a) We applied GATK v.3.8 “RealignerTargetCreator” and “IndelRealigner” modules to reduce false‐positive SNPs where alignment error occurs across overlapping reads; (b) GATK v.4.0.2.1 HaplotypeCaller module and SAMtools mpileup (v1.3.1) command were then used to detect SNPs and indels; (c) after that, we used GATK v.4.0.2.1 “SelectVariants” module to select the same variation shared by “HaplotypeCaller” and SAMtools; and (d) we then applied GATK v.4.0.2.1 “VariantFiltration” module to filter bad variants with strict parameters recommended from the GATK Best Practices (Van der Auwera et al., 2013): QD < 10.0 || MQ < 50.0 || FS > 10.0 || MQRankSum < −5.0 || ReadPosRankSum < −8.0; (e) repeat the above steps 2 and 3 until the variations were convergent, and the generated variations were defined as known dbSNP dataset. With the generated dbSNP dataset, we applied GATK v.4.0.2.1 “BaseRecalibrator” and “ApplyBQSR” modules to generate quality scores recalibrated bam files for every individual. Finally, we applied GATK v.4.0.2.1 “HaplotypeCaller” module to separately detect variation from the bam file generated from BQSR process for each individual.

After obtaining the genotype calls from the GATK's best practice pipeline, we applied several data quality filters to control the data quality. We first applied the GATK v.4.0.2.1 “SelectVariants” module to split the variants into SNVs and indels from the generated VCF format files. Then, we used the hard filter module GATK v.4.0.2.1 “VariantFiltration” to exclude false‐positive variant sites with the following parameters (Liu et al., 2018; Van der Auwera et al., 2013): for SNVs (QD < 2.0 || MQ < 40.0 || FS > 60.0 || MQRankSum < −12.5 || ReadPosRankSum < −8.0); for indels (QD < 2.0 || FS > 200.0 || ReadPosRankSum < −20.0 || SOR > 10.0). Single‐nucleotide variants (SNVs) and indels with much lower or higher read depth than expected were removed because lower depth and large copy‐number variation might lead to overcalling and miscalling of variants. We set the minimum and maximum read depths (DP value) for variant calling as 0.1‐fold and twofold of the average depth of sequencing, respectively (Wang et al., 2017). Additionally, we also filtered the sites that were an “N” in the reference genome.

We downloaded the genome annotation file in gff3 format and protein and transcript files in fasta format of *B. belcheri* v.18h27 and *B. floridae* v.2.0 genomes from the NCBI Genome Database. After removing duplicate records, pseudogenes, noncoding RNA genes, and genes without a transcript ID, the final annotation files for *B. belcheri* v.18h27 and *B. floridae* v.2.0 contained 25,063 and 28,621 genes, respectively. The final genome annotation files were then used in ANNOVAR v.2016‐02‐01 software (Wang, Li, & Hakonarson, 2010) and SNPeff v.4.3 software (Cingolani et al., 2012) with default settings to classify the called variants into intergenic regions, untranslated regions (UTRs), upstream and downstream regions (within 1 kb of a transcription start or end site), intronic regions, splicing sites (within 2 bp of a splicing junction), and exonic regions. The SNVs located in exonic regions were further classified into nonsynonymous, synonymous, stop‐codon gain, and stop‐codon loss variants. The indels located in exonic regions were further classified into nonframeshift, frameshift, stop‐codon gain, and stop‐codon loss variants.

### 2.5 Inference of demographic history using PSMC

We applied the Pairwise Sequentially Markovian Coalescent (PSMC) method developed by Li and Durbin (2011) to infer the trajectory of the demographic history across time for Chinese and American lancelets (Li & Durbin, 2011). PSMC analysis requires a whole‐genome diploid consensus sequence in fastq format. Considering the significant influence of different sequencing depths, generation times, and per‐generation mutation rates of species, we used different settings to optimize the time estimation of the PSMC results. To evaluate the influence of sequencing depth for PSMC results, we used the SAMtools view command to subsample the high‐depth samples with the following parameters: samtools view –bs FLOAT (integer part as seed). We then presented PSMC plots of 15×, 20×, 25×, 30×, 35×, 40× reads for these high‐depth samples (BB_ZJ1, BB_XM1, BF_CN1, and BF_TP1), respectively. For each of these samples, we obtained the consensus sequences using the SAMtools mpileup command and the BCFtools call command with the following parameters: “samtools mpileup ‐C50 ‐q25 ‐uf *.fa (reference genome) *.bam | bcftools call ‐c ‐ | vcfutils.pl vcf2fq ‐d minDP ‐D maxDP> ∗.psmc.fq”. To minimize the impact of collapsed regions in the whole‐genome, we excluded all sites for which read depth was less than one‐third of the average read depth and more than twice the average read depth across the genome using the parameters –d and –D. After that, over 75% of the genome are reserved for the downstream PSMC analysis of all *B. belcheri* and *B. floridae* individuals. We then used the program fq2psmcfa integrated into the PSMC software to transform the consensus sequence (*.psmc.fq) into the fasta‐like format files for downstream analysis. The program “psmc” was used to infer the demographic histories from the individual genomes, and the settings were chosen manually according to suggestions given by Li and Durbin (Li & Durbin, 2011). The maximum number of iterations was set to 25 by the –N option, the initial theta/rho ratio value to 5 by the ‐r option, and the upper limit of the most recent common ancestor (TMRCA) was first set to 13 by the ‐t option. The effective population size was inferred across 61 free atomic time intervals by the ‐p option: "1*2 + 58*1 + 2*2", which means that the first population size parameter spans the first two atomic time intervals, each of the next 58 parameters spans one interval, and the last two parameters span two intervals, respectively. We used the program psmc_plot.pl to visualize the trajectory of demographic history across time for every individual.

The time estimation of demographic history can be significantly influenced by the mutation rate and generation time. We estimated the generation time to be 1.5 years for *B. belcheri* and 1 year for *B. floridae* based on previous studies (Igawa et al., 2017; Theodosiou et al., 2011; Yu & Holland, 2009). In this study, all the results assume that the generation time in modern *Branchiostoma* is representative of the ancestral population. The per‐generation mutation rate is another important factor for the time estimation of demographic history. Previous studies have documented that the per‐generation mutation rate scales inversely with genome size in DNA‐based microbes, but scales positively in cellular organisms (Drake, 1991; Lynch, 2010). The best fitting model for per‐generation mutation rate was 2.585324 × 10^−10^ × g^0.584^ for genome size g in Mb (Crellen et al., 2016; Lynch, 2010), giving the estimates of 9 × 10^−9^ for *B. belcheri* and 1 × 10^−8^ for *B. floridae*. Previous studies of amphioxus genome projects have estimated the mutation rates of *B. belcheri* and *B. floridae* to be on the order of 10^−9^ to 10^−8^ substitutions/site/generation (Huang et al., 2014; Putnam et al., 2008). To account for the potential bias of mutation rates, we also performed demographic analysis with other two mutation rates (per site per generation) for *B. belcheri* and *B. floridae*: 3 × 10^−9^ and 6 × 10^−9^. We compared the PSMC results of different mutation rates and discussed their corresponding outcomes in the results section. Finally, we performed 100 bootstrap replicates to assess uncertainty in effective population size estimates for each individual. Bootstrapping was conducted by randomly sampling with replacement 5‐Mb sequence segments obtained from the consensus genome sequence using the splitfa script provided with the PSMC software.

## 3 RESULTS

### 3.1 Individual‐level genome sequencing and reads mapping

Five wild *B. belcheri* individuals (BB_ZJ1, BB_ZJ2, BB_XM3, BB_XM4, and BB_XM5) and five laboratory‐raised *B. floridae* individuals (BF_CN1, BF_CN2, BF_CN3, BF_CN4, and BF_CN5) were selected for whole‐genome resequencing. Genomic DNA was extracted from their muscular tissues and whole‐genome resequencing was then performed using the Illumina HiSeq platform. After stringent quality filtering, we generated a total of 151.72 Gbp of clean reads for five wild *B. belcheri* individuals and 124 Gbp of clean reads for five laboratory‐raised *B. floridae* individuals (Table 1). The clean reads were submitted to the NCBI Short Read Archive under BioProject accession number PRJNA573992. The other short read sequences of five *B. belchri* and five *B. floridae* individuals were downloaded from NCBI. The clean reads of ten *B. belcheri* individuals were then mapped to the *B. belcheri* v.18h27 reference genome, and the clean reads of ten *B. floridae* individuals were mapped to the *B. floridae* v.2.0 reference genome. The genome sizes of *B. belcheri* v.18h27 reference genome are 426.12 Mbp, including 2,307 scaffolds and 25,063 genes, and only 1.3% of the whole genome (5,528,395 sites) were marked as “N” (Huang et al., 2014). The genome size of *B. floridae* v.2.0 reference genome was 521.9 Mbp, including 398 scaffolds and 28,621 genes, and about 8% (41,496,865 sites) of the genome were marked as “N” (Putnam et al., 2008). The minimum mapping rate was 90.46% for BF_TP5, the maximum was 96.67% for BB_XM4, and the average was 93.58% for all twenty *Branchiostoma* individuals (Table 1). The minimum effective genome‐wide depth was 11.48× for BF_TP4, and the maximum was 122.87× for BB_ZJ1. Additionally, we also surveyed the effective genome coverage for all *Branchiostoma* individuals. Additionally, the effective genome coverages of *B. belcheri* (average coverage: 90.44%) were slightly higher than those of *B. floridae* (average coverage: 88.48%) (Table 1).

**TABLE 1:** Description of mapping results of twenty *Branchiostoma* individuals used in this study

Sample	Clean reads	Size (Gbp)	Mapping rate (%)	Effective depth (X)	Genome coverage > 1X (%)
BB_ZJ1	417,569,456	62.64	94.69	122.87	92.60
BB_ZJ2	366,229,340	54.93	94.51	107.57	92.30
BB_ZJ3	210,710,342	21.07	92.99	42.30	90.90
BB_ZJ4	219,989,476	22.00	92.49	43.84	91.40
BB_ZJ5	234,033,972	23.40	92.33	46.53	91.10
BB_XM1	125,955,488	18.89	96.17	38.32	89.90
BB_XM2	129,206,312	19.38	96.03	39.35	90.00
BB_XM3	75,230,716	11.28	96.60	22.69	88.70
BB_XM4	78,312,136	11.75	96.67	23.63	88.80
BB_XM5	74,100,078	11.12	95.97	22.17	88.70
BF_CN1	521,642,060	78.25	91.33	119.93	90.90
BF_CN2	74,389,546	11.16	92.86	17.29	86.90
BF_CN3	77,936,894	11.69	92.59	18.12	88.40
BF_CN4	74,198,594	11.13	92.14	17.06	88.20
BF_CN5	78,495,986	11.77	91.73	18.02	86.90
BF_TP1	159,308,674	39.83	95.00	59.60	89.20
BF_TP2	148,445,394	37.11	94.60	55.65	89.20
BF_TP3	66,493,198	9.97	91.06	15.06	88.80
BF_TP4	50,718,342	7.61	91.31	11.48	87.60
BF_TP5	57,310,178	8.60	90.46	12.88	88.70

### 3.2 Detection and annotation of genetic variations in *B. belcheri* and *B. floridae*

**FIGURE 2:**
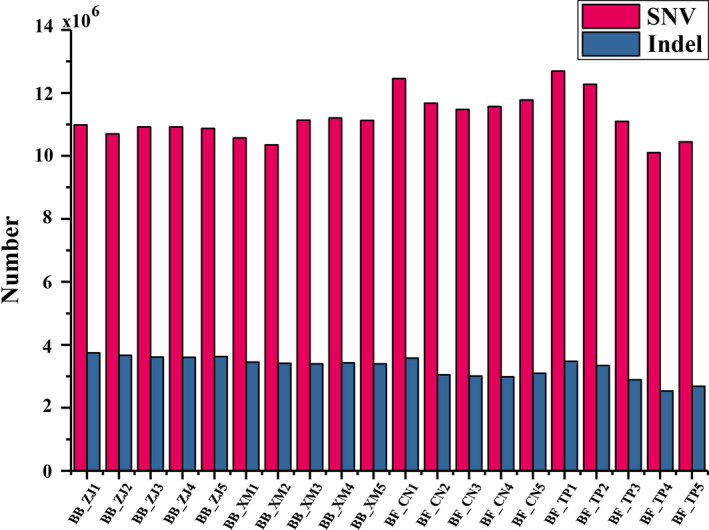
Distribution of SNVs and indels in different *Branchiostoma* individuals. The number of SNVs and indels are shown in red and blue, respectively

**FIGURE 3:**
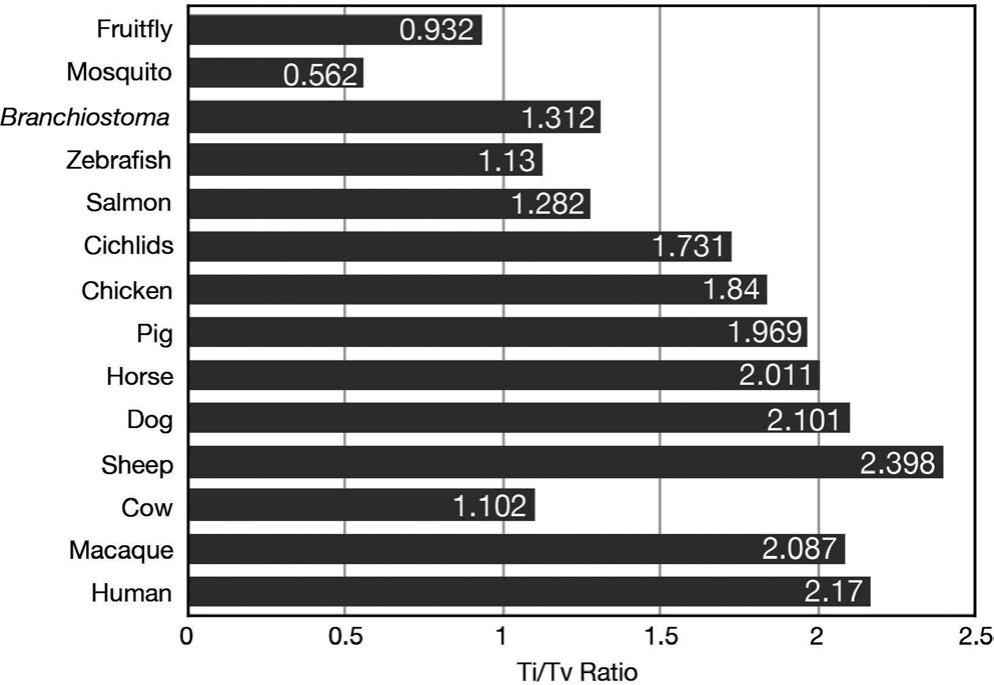
Comparison of Ti/Tv ratios in 14 different species. The Ti/Tv ratios of each species were calculated from their whole‐genome SNP data (VCF format), which were downloaded from the NCBI SNP database (https://ftp.ncbi.nih.gov/snp/organisms/archive/)

The alignment files of each individual were further processed with our customized genotyping pipeline to produce the genotype calls. Then, we applied stringent variant filtrations to further control the genotype calls (Liu et al., 2018; Moran et al., 2019). As shown in Table S1, an average of 12.82% false‐positive variants was filtered in ten *B. belcheri* individuals and an average of 11.44% was filtered in ten *B. floridae* individuals. After filtering false‐positive variants and large indels (>50 bp), we generated the final variants for each *Branchiostoma* individual. As shown in Table 2 and Figure 2, the number of variants in *B. belcheri* was relatively stable, probably because the sequencing depths were greater than 20× for all *B. belcheri* individuals. However, the number of variants varied widely in *B. floridae*, showing that the higher the sequencing depth, the more variants detected. To further quantify the genome‐wide heterozygosity, we calculated the ratio of heterozygous variants against all genomic sites. The results showed that five *B. belcheri* individuals captured from Xiamen Bay, China, had lower heterozygosity of ~2.04%, while the other five *B. belcheri* from Zhanjiang, China, had higher heterozygosity (average: 2.15%). Additionally, we found the heterozygosity of low‐depth individuals (BF_CN2, BF_CN3, BF_CN4, BF_CN5, BF_TP3, BF_TP4, and BF_TP5) was lower than that of high‐depth individuals (BF_CN1, BF_TP1, and BF_TP2), indicating the failure to call heterozygous sites with low‐depth data. The transitions/transversions (Ti/Tv) ratios in twenty *Branchiostoma* individuals ranging from 1.2966 in BB_XM1 to 1.3170 in BF_TP2 (Figure 3, Table S2).

**TABLE 2:** Summary of heterozygosity in 20 *Branchiostoma* individuals used in this study

Sample	Depth (X)	Heterozygous variants		Homozygous variants		Total	Heterozygosity (%)
		SNV	InDel	SNV	InDel		
BB_ZJ1	122.87	6,667,424	2,513,574	4,315,443	1,234,208	14,730,649	2.18
BB_ZJ2	107.57	6,468,244	2,440,565	4,232,815	1,227,381	14,369,005	2.12
BB_ZJ3	42.30	6,691,021	2,386,344	4,232,195	1,222,112	14,531,672	2.16
BB_ZJ4	43.84	6,678,417	2,383,179	4,244,807	1,222,405	14,528,808	2.15
BB_ZJ5	46.53	6,662,863	2,397,469	4,211,658	1,227,247	14,499,237	2.15
BB_XM1	38.32	6,345,815	2,272,753	4,221,585	1,180,991	14,021,144	2.05
BB_XM2	39.35	6,189,822	2,231,289	4,157,077	1,180,801	13,758,989	2.00
BB_XM3	22.69	6,491,287	2,157,883	4,641,148	1,241,488	14,531,806	2.06
BB_XM4	23.63	6,555,228	2,185,255	4,651,625	1,240,193	14,632,301	2.08
BB_XM5	22.17	6,465,479	2,148,918	4,660,332	1,244,673	14,519,402	2.05
BF_CN1	119.93	7,327,181	2,332,127	5,131,042	1,250,744	16,041,094	2.01
BF_CN2	17.29	6,349,470	1,796,747	5,320,095	1,248,404	14,714,716	1.70
BF_CN3	18.12	6,269,043	1,780,006	5,203,018	1,226,832	14,478,899	1.68
BF_CN4	17.06	6,473,810	1,810,056	5,098,363	1,178,484	14,560,713	1.72
BF_CN5	18.02	6,493,887	1,849,549	5,278,909	1,244,416	14,866,761	1.74
BF_TP1	59.60	7,379,613	2,234,389	5,315,412	1,242,830	16,172,244	2.00
BF_TP2	55.65	7,099,422	2,130,058	5,171,115	1,210,348	15,610,943	1.92
BF_TP3	15.06	6,105,547	1,725,098	4,986,421	1,163,420	13,980,486	1.63
BF_TP4	11.48	5,148,738	1,401,908	4,958,838	1,134,381	12,643,865	1.36
BF_TP5	12.88	5,606,746	1,566,891	4,834,706	1,119,927	13,128,270	1.49

Based on the genomic annotations from the reference genomes, we divided the total genetic variations into different regions, including intronic regions, intergenic regions, exonic regions, upstream or downstream regions, untranslated regions, and splicing sites (Figure S1, Tables S2 and S3). To further evaluate the functional effect of SNVs in the exonic regions, we divided the exonic SNVs into nonsynonymous, synonymous, stop‐codon gained, and stop‐codon loss SNVs. Similarly, we divided the exonic indels into frameshift, nonframeshift, stop‐codon gained, and stop‐codon loss indels. The highest occurrence of exonic indels corresponded to double base pairs and the second highest to single base pairs (Figure S2). Except for the first three nucleotides window, there were more nonframeshift indels (multiples of three nucleotides) than frameshift indels in each of the three nucleotides window, probably because the nonframeshift indels consist of a multiple of three base pairs, thus introducing an insertion or deletion of one or more amino acids while keeping the rest of the protein sequence unchanged.

### 3.3 Estimation of generation time for *Branchiostoma*

As previously discussed, the growth rate of *Branchiostoma* varies in different species, thus contributing to different generation times of species (Chen, Shin, & Cheung, 2008; Stokes & Holland, 1995). The body‐length ranges of one‐, two‐, and three‐year‐old *B. belcheri* were estimated to be 5–28, 28–38, and 38–45 mm, respectively (Chen et al., 2008). In contrast, the Florida lancelet *B. floridae* has a rapid growth rate and its body length was observed to be over 30 mm within only one year (Stokes & Holland, 1995, 1996). As described in previous studies, the minimum body length at sexual maturity is about 25–28 mm for *B. belcheri* and the mature *B. belcheri* individuals mainly belong to 1‐ and 2‐year‐old groups (Chen et al., 2008). In contrast, the Florida lancelet *B. floridae* has a rapid growth rate and its body length was observed to be over 30 mm within only 1 year. As described in previous studies, the minimum body length at sexual maturity is about 25–28 mm for *B. belcheri* and the mature *B. belcheri* individuals mainly belong to 1‐ and 2‐year‐old groups. Stokes and Holland (1995, 1996) reported that the minimum mature individual of *B. floridae* had a body length of about 23 mm body and the mature individuals mainly belonged to the one‐year‐old group (Stokes & Holland, 1995, 1996). From the above, we estimated a generation time of 1.5 years for *B. belcheri* and 1 year for *B. floridae* to infer the ancestral *N*_e_ dynamics in the following PSMC analyses.

### 3.4 Influence of sequencing depth on PSMC analysis

**FIGURE 4:**
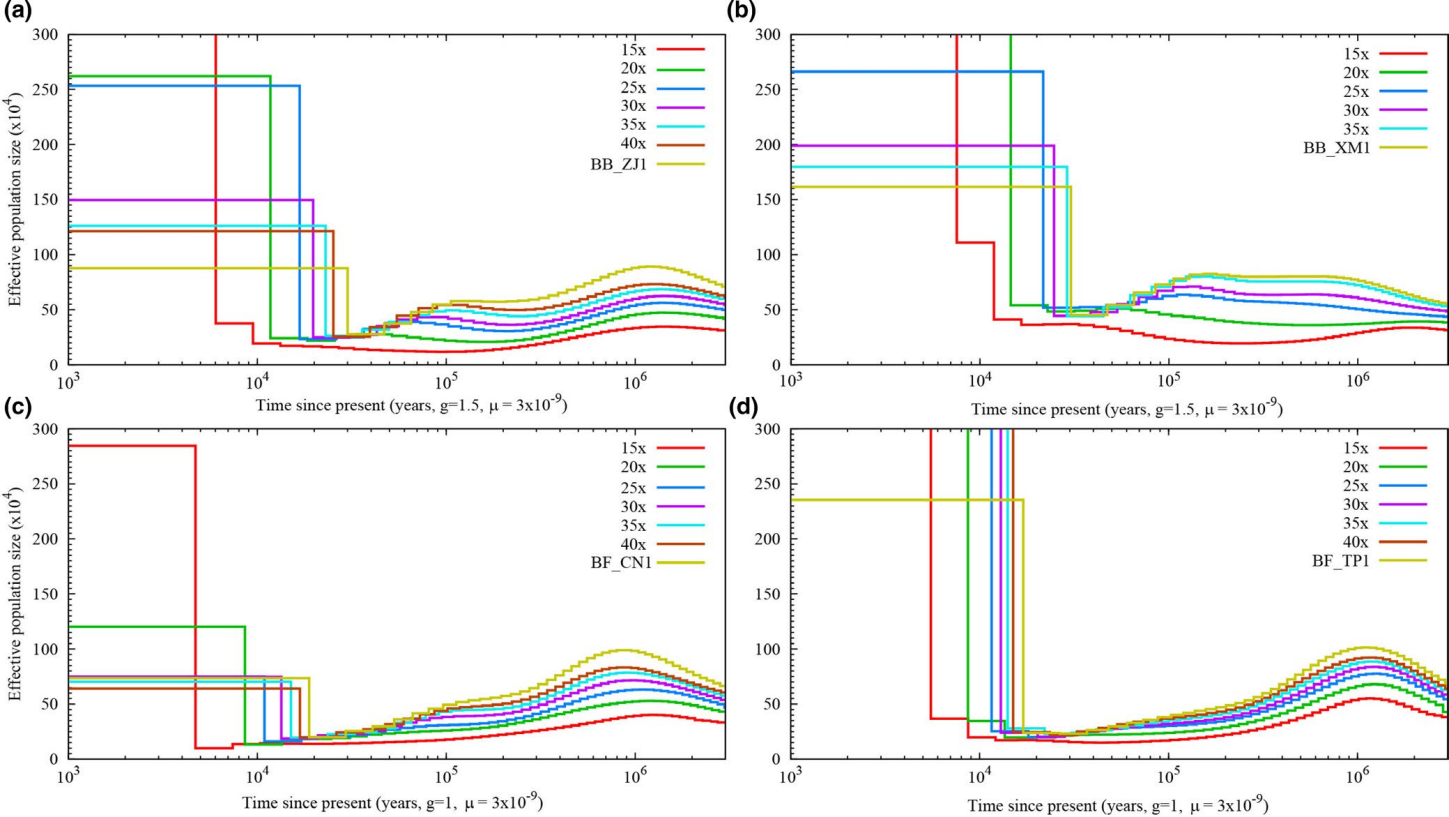
PSMC results for different sequencing depth of four *Branchiostoma* individuals. The sequencing depth of each individual is shown in the figure legend of top‐right corner. The numbers of years per generation (g) for *B. belcheri* and *B. floridae* are 1.5 and 1, respectively. The mutation rate per generation (μ) for *B. belcheri* and *B. floridae* is 3 × 10^−9^

As our data included individuals for which the mean sequencing varied from 11.48× to 122.87× (Table 1), we first need to investigate the influence of the combined effect of sequencing depth and genome coverage on the results of the PSMC analysis. To perform such an evaluation, we presented the PSMC plots of 15×, 20×, 25×, 30×, 35×, 40× reads for BB_ZJ1, BB_XM1, BF_CN1, and BF_TP1, respectively. A similar shape of the *N*_e_ curve was seen in each individual with different depths, although amplitudes were less pronounced at low depth (Figure 4), most probably due to the failure to call heterozygous sites with low‐depth data. The amplitudes of PSMC curves are proportional to the sequencing depth, and the higher the depth, the greater the amplitude. Additionally, we also found that the minimum *N*_e_ estimation and the timing of *N*_e_ were related to the sequencing depth. The minimum *N*_e_ and its timing were no longer drastically changed until the sequencing depth ≥25× in all selected *B. belcheri* and *B. floridae* individuals. In other words, the population size dynamics of *Branchiostoma* were remarkably similar among individuals if the sequencing depth over 25×, suggesting that the sequencing depth should be used as filtering thresholds in PSMC analysis. Based on these results, we discussed the population size dynamics of *B. belcheri* and *B. floridae* populations by only considering individuals that have a mean sequencing depth ≥25×.

### 3.5 Population size dynamics of *Branchiostoma* during the Pleistocene

**FIGURE 5:**
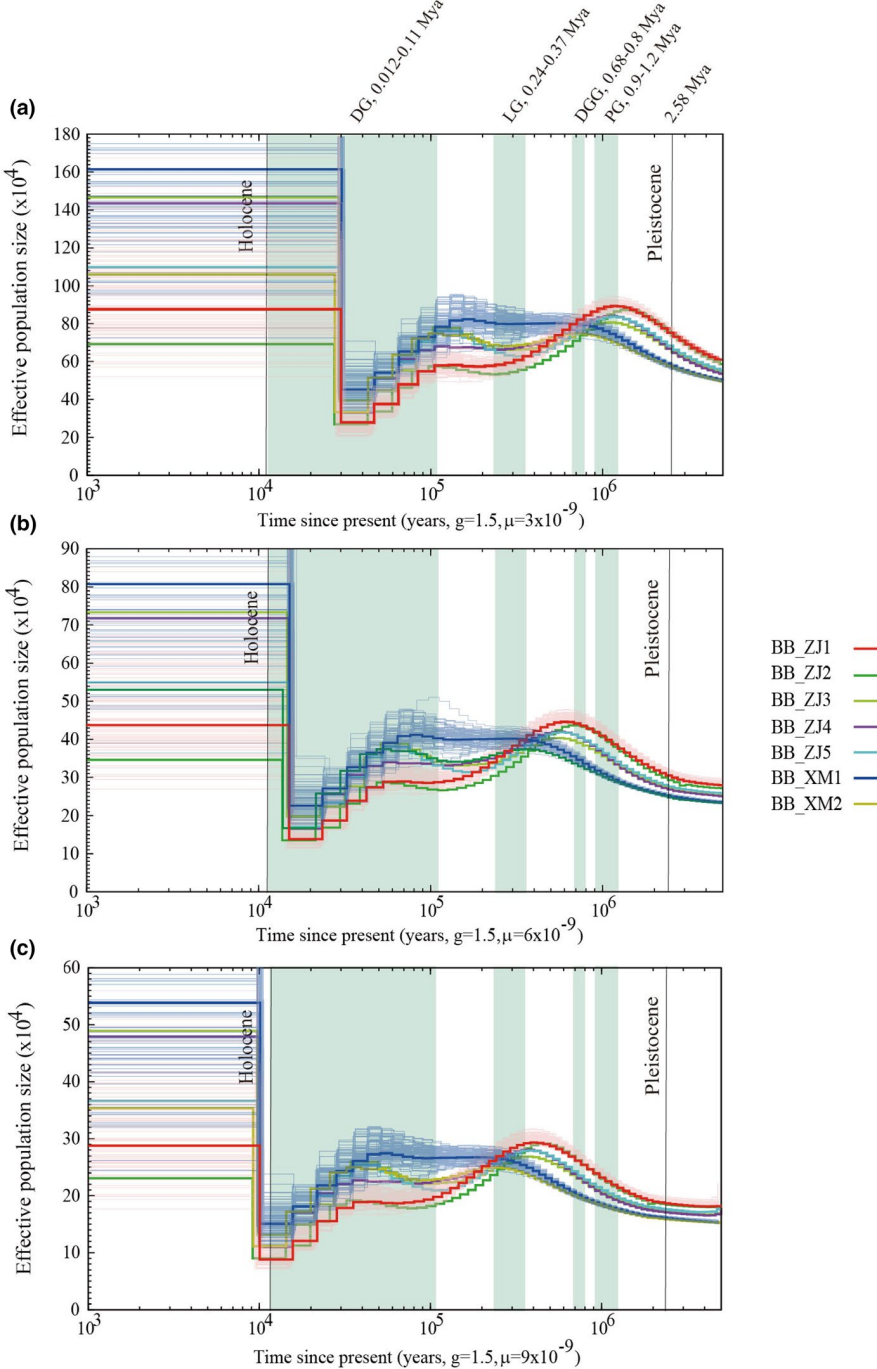
Temporal dynamics of effective population size for seven high‐depth *B. belcheri* individuals. (a) The generation time (g) and the mutation rate (μ) are assumed to be 1.5 years and 3 × 10^−9^, respectively. (b) The generation time (g) and the mutation rate (μ) are assumed to be 1.5 years and 9 × 10^−9^, respectively. The four light green areas indicate four major Pleistocene glaciations in Eastern China. The bold curves show the estimates based on original data, and the light curves show the estimates for 100 bootstrapped sequences

To better understand the ancestral temporal dynamics of *B. belcheri* and *B. floridae* populations during the Pleistocene, we applied the PSMC method to estimate the ancestral effective population sizes of two *B. belcheri* populations from Zhanjiang and Xiamen, China, and two *B. floridae* populations from Fuzhou, China and Tampa Bay, USA. With an assumed per‐generation mutation rate of 3 × 10^−9^ and generation time of 1.5 for *B. belcheri*, the PSMC results revealed different *N*_e_ trajectories between Zhanjiang and Xiamen *B. belcheri* populations (Figure 5a). During the early Pleistocene, the Zhanjiang *B. belcheri* population experienced a continuous expansion and reached its first population peak (Mean *N*_e_: ~900,000) approximately 1.2 Mya. After that, they suffered from a long‐term decline from 1.2 Mya to 0.24 Mya and then remained at a low level (Mean *N*_e_: ~560,000) over the next 100,000 years. A population bottleneck of *N*_e_ ~ 300,000 was reached about 30 kya, followed by a population expansion occurred during the late Pleistocene. The temporal dynamics of the *B. belcheri* population from Xiamen differed from that from Zhanjiang both in terms of the shape of the *N*_e_ curve and in the consistency among the populations. The Xiamen *B. belcheri* population experienced a very long‐term expansion from *N*_e_ ~ 580,000 at 2.58 Mya to *N*_e_ ~ 760,000 at 0.68 Mya. Then, the Xiamen population experienced a cycle of slow population contraction and expansion and was stable during the middle Pleistocene. During the late Pleistocene, the Xiamen *B. belcheri* population shared the same pattern of temporal dynamics with the Zhanjiang population. When applying higher alternative mutation rates of 6 × 10^−9^ and 9 × 10^−9^ to infer the demographic history of *B. belcheri* population, the changes in inferred effective population size are shifted toward more recent times, and the overall estimates of *N*_e_ are lowered to fluctuate between ~90,000 and ~440,000.

**FIGURE 6:**
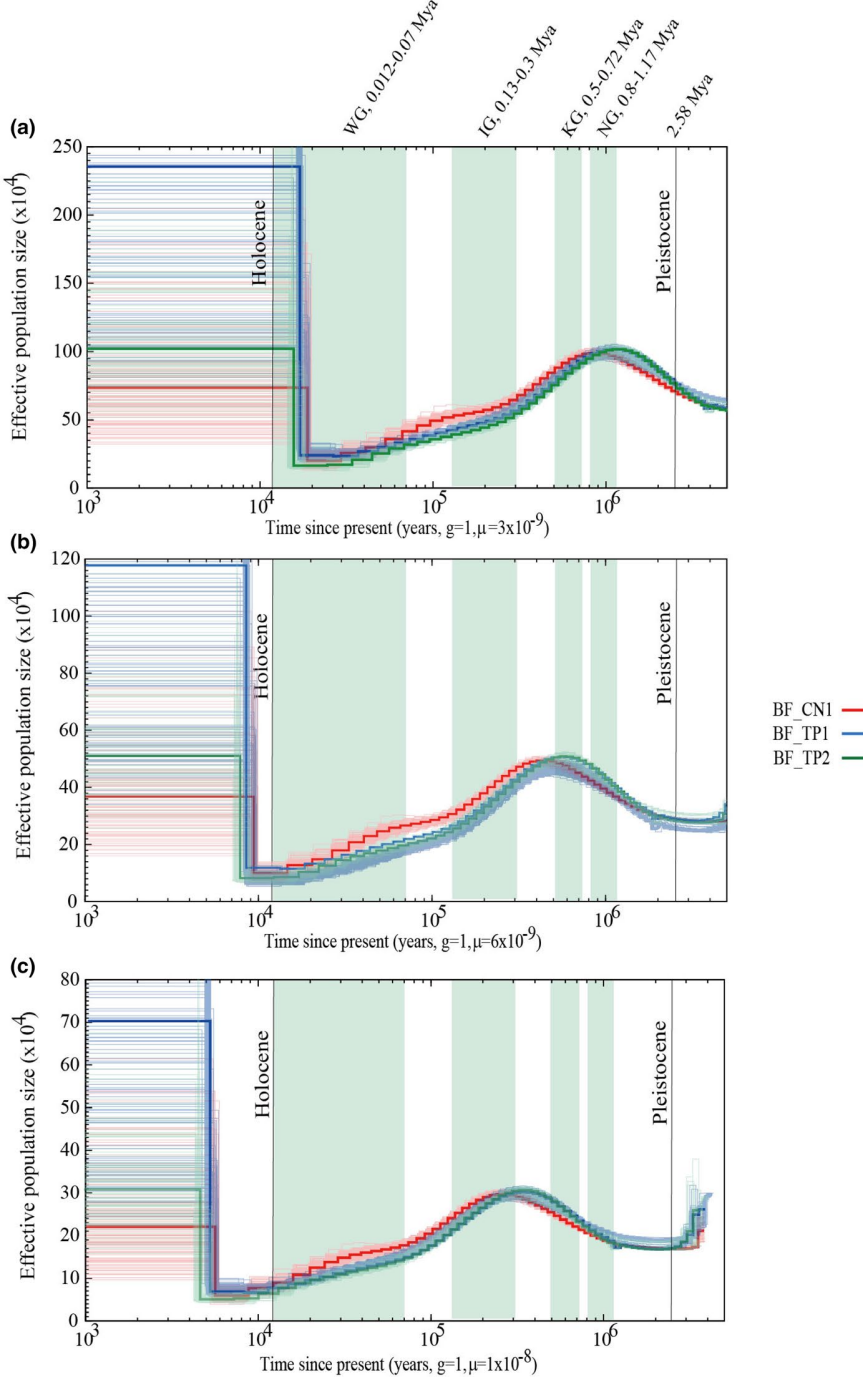
Temporal dynamics of effective population size for three high‐depth *B. floridae* individuals. (a) The generation time (g) and the mutation rate (μ) are assumed to be 1 year and 3 × 10^−9^, respectively. (b) The generation time (g) and the mutation rate (μ) are assumed to be 1 year and 1 × 10^−8^, respectively. The four light green areas indicate four major Pleistocene glaciations in North America. The bold curves show the estimates based on original data, and the light curves show the estimates for 100 bootstrapped sequences

The PSMC curves of *B. floridae* indicate that all the *B. floridae* individuals shared ancestral demographic history for most of the Pleistocene period (Figure 6). With an assumed per‐generation mutation rate of 3 × 10^−9^ and generation time of 1 for *B. floridae*, the *B. floridae* population size increased from *N*_e_ ~ 750,000 at 2.58 Mya to a population peak of *N*_e_ ~ 1,000,000 at 1.17 Mya. Then, the *N*_e_ of *B. floridae* went through a sustained contraction with a population bottleneck of *N*_e_ ~ 200,000 at about 20 kya, followed by a population expansion occurred during the late Pleistocene. When applying higher alternative mutation rates of 6 × 10^−9^ and 1 × 10^−8^ for *B. floridae* population, the changes in inferred effective population size are shifted backward in time, and the overall estimates of *N_e_* are lowered to fluctuate between ~50,000 and ~510,000.

### 3.6 Cycles of population expansions and contractions of *Branchiostoma* during the Pleistocene

Many species suffered from extreme contraction during the glacial period, but re‐expanded during the interglacial period. During the Quaternary period, there were four main glacial periods in Eastern China, including the Dali Glaciation (DG, ~0.015–0.11 Mya), Lushan Glaciation (LG, ~0.24–0.37 Mya), Da Gu Glaciation (DGG, ~0.68–0.8 Mya), and Poyang Glaciation (PG, ~0.9–1.2 Mya) (Derbyshire, 1987; Severinghaus & Brook, 1999; Zhou, Li, Zhao, Jie, & Zheng, 2011). Similarly, corresponding glaciations with different traditional regional glacial names were also found in North America, Europe, and the Alpine region (Johnson, 1986; Lisiecki & Raymo, 2005; Richmond & Fullerton, 1986).

As shown in Figure 5a, when applying the mutation rate of 3 × 10^−9^ for *B. belcheri* population, both of the Zhanjiang and Xiamen populations experienced a very long‐term population expansion before the first glaciation of the early Pleistocene, but then, the two populations showed different patterns of *N*_e_ dynamics during the middle Pleistocene. From the beginning of PG, the *N*_e_ of the Zhanjiang *B. belcheri* population started to decline and this trend continued until the end of the third glaciation of the Pleistocene. Then, the Zhanjiang population maintained a stable *N_e_* during the interglacial period between the third and last glaciations. In contrast, the *N*_e_ of Xiamen *B. belcheri* population slowly increased during the first glaciation of the Pleistocene and remained high from the end of the first glaciation to the start of the last glaciation of the Pleistocene (LGP). Both of the *B. belcheri* populations from Chinese seawaters then suffered from an abrupt decline in *N*_e_ at the beginning of the LGP and contributed to a population bottleneck at about 30 kya. Subsequently, a population expansion took place within a relatively short period of time during the late Pleistocene. However, when applying higher alternative mutation rates of 6 × 10^−9^ and 9 × 10^−9^ for *B. belcheri* population, both of the Zhanjiang and Xiamen populations experienced a very long‐term population expansion during the first two glaciations of early Pleistocene (PG and DGG) (Figure 4b,c), and the expansion with mutation rate of 9 × 10^−9^ continued until the arriving of the third glaciation (LG, 0.24–0.37 Mya) (Figure 5c). From the beginning of LG to the early DG, the Zhanjiang population suffered from a long‐term reduction in *N*_e_, while the *N*_e_ of Xiamen population remained at a very high level in this stage. Both of the Zhanjiang and Xiamen *B. belcheri* populations suffered from an abrupt decline in *N*_e_ at the early or middle of the LGP. For the mutation rate of 6 × 10^−9^, the abrupt decline took place at ~70 kya and contributed to a population bottleneck at ~21 kya. For the mutation rate of 9 × 10^−9^, the decline was shifted toward more recent times (~40 kya) and contributed to the lowest estimate of *N*_e_ corresponding to a population bottleneck at ~13 kya.

The historical *N*_e_ curves of the *B. floridae* population show subtly different patterns with *B. belcheri* during the Pleistocene (Figure 6). With an assumed per‐generation mutation rate of 3 × 10^−9^ for *B. floridae*, the *B. floridae* population experienced a similar population expansion to that of Chinese *B. belcheri* during the early Pleistocene (Figure 6a). Subsequently, *B. floridae* suffered from a long‐term population reduction during the next 1.1 million years and reached a population bottleneck at about 20 kya. The *B. floridae* population experienced a fivefold decline in *N*_e_ since the first glaciation of the Pleistocene. Strikingly, the *B. floridae* population from American seawaters experienced a population expansion during the late Pleistocene, which was also seen in the two *B. belcheri* populations from Chinese seawaters (Figures 4 and 5). Within a very short time, the effective population sizes of *Branchiostoma* almost recovered or exceeded their population peaks during the early and middle Pleistocene. When applying higher alternative mutation rates of 6 × 10^−9^ and 1 × 10^−8^ for *B. floridae* population, the *N*_e_ of *B. floridae* population experienced a very long‐term expansion during the first two glaciations (NG and KG) at the beginning of the Pleistocene, and the expansion with 1 × 10^−8^ continued until the arriving of the third glaciation (IG, 0.13–0.3 Mya). Then, the *B. floridae* population suffered from a very long‐term population reduction until the most recent time and reached a population bottleneck at about 10 kya.

## 4 DISCUSSION

The ratio of the number of transitions to the number of transversions (Ti/Tv) for a pair of sequences becomes 0.5 when there is no bias toward either transitional or transversional substitution. The Ti/Tv ratio of *Branchiostoma* was ~1.3, which was much less than the criterion of many mammal genomes, but a little higher than that of some fishes (DePristo et al., 2011; Lachance et al., 2012). Although transversion mutations (A ↔ C, A ↔ T, C ↔ G, and G ↔ T) should be twice as much as transition mutations (A ↔ G and C ↔ T), transition mutations are more likely than transversions because substituting a single ring structure for another single ring structure is more likely than substituting a double ring for a single ring. As shown in Figure 3, the Ti/Tv ratio ranged from the lowest of 0.562 in mosquito to the highest of 2.389 in sheep. The extremely low Ti/Tv ratio of *Branchiostoma* population most probably resulted from the very low levels of CpG methylation in its genome, which has been proved by a recent publication (Marlétaz et al., 2018). In human and some other mammal genomes, CpG methylation is a very common characterized epigenetic modifications that cells use to control gene expression, and Cytosine (C) on the genome is prone to C ‐> T transitions under methylation modification (Cooper, Mort, Stenson, Ball, & Chuzhanova, 2010; Li & Zhang, 2014). Additionally, transitions in genomes are less likely to result in amino acid substitutions (due to wobble base pair) and are therefore more likely to persist as synonymous SNVs in populations.

The PSMC approach is an excellent method to infer detailed ancestral population size dynamics over time using information from whole‐genome sequences and has recently been used in many genome projects. However, as described by Mazet et al. (Chikhi et al., 2018; Mazet, Rodriguez, Grusea, Boitard, & Chikhi, 2016), when considering structured populations, the population structure can mimic the signature of any demographic scenario, because PSMC assumes a panmictic model and the gene flow patterns could result in the changes of estimated *N*_e_ (Chikhi et al., 2018; Gautier et al., 2016). Therefore, it is not possible to distinguish between a scenario involving habitat fragmentation and a scenario of actual population size changes from the PSMC analyses. It also should be noted that the inferred *N*_e_ over time largely depends on the sequencing depth and the parameters used in the PSMC analysis (Li & Durbin, 2011; Nadachowska‐Brzyska, Burri, Smeds, & Ellegren, 2016). As described by Nadachowska‐Brzyska et al. (2016), an appropriate sequencing depth for proper inference of demographic histories using PSMC is ≥18×. However, we found the threshold (18×) of sequencing depth was not enough to get a proper PSMC curve for *Branchiostoma*. The best threshold of sequencing depth for *Branchiostoma* was ≥25× (Figure 4), which most probably due to its high heterozygosity to fail call heterozygous sites with low‐depth data (Figures S3 and S4).

The recombination rate, per‐generation mutation rate, and generation time estimates are three important parameters for the PSMC results, and the inference of ancestral *N*_e_ can be biased if they are under‐ or overestimated (Nadachowska‐Brzyska et al., 2015, 2016). The recombination rate will not influence the accuracy of PSMC results, when it was set appropriately. As shown in Figure S5, the PSMC results with different recombination rates are very consistent during the most of the Pleistocene. However, the estimated *N*_e_ in the most recent past is quite different, because few recombination events are left in the present sequence, which reduces the power of PSMC. Additionally, the per‐generation mutation rate and generation time of species influence the PSMC results in a predictable manner. For example, when given a fixed mutation rate per year, a doubling of the generation time will move the PSMC curve forward in time but does not change the *N*_e_ (Figure S6); however, when given a fixed generation time, a doubling of the per‐generation mutation rate will halve the estimate of *N*_e_ and also move the PSMC curve backward in time (Figure S7). In this study, we assumed that genetic variation in modern *Branchiostoma* species is representative of the ancestral population. Based on recent studies about amphioxus growth and gonad development, we estimated the generation time to be 1.5 years for *B. belcheri* and 1 year for *B. floridae* to infer the ancestral *N*_e_ dynamics (Stokes & Holland, 1995, 1996). Additionally, previous studies have found that the growth and gonadal development of *Branchiostoma* are closely related to seawater temperature, and the higher the seawater temperature, the faster the growth and the earlier the gonadal development (Igawa et al., 2017; Theodosiou et al., 2011; Yu & Holland, 2009). Therefore, the effective population size of *Branchiostoma* should decline during the most glacial periods, especially during the last maximum glaciation. As shown in Figures 5 and 6, only the effective population size generated by a per‐generation mutation rate of 3 × 10^−9^ suffered from a large reduction during glacial periods and remained stable during the interglacial periods of the Pleistocene. Therefore, the per‐generation mutation rate of 3 × 10^−9^ is more likely to represent the changes in the historical effective population sizes over time than the other two higher mutation rates.

The PSMC approach has recently been applied to infer demographic history in many genome projects but has never been applied to Cephalochordate. The Quaternary Period spans from approximately 2.58 million years ago (Mya) to the present, which can be divided into two epochs: the Pleistocene (2.58 Mya to 11.7 kya) and the Holocene. During the Quaternary Period, global climatic fluctuations could influence the demography and distribution of biodiversity on Earth. Using the PSMC approach, we inferred the ancestral *Ne* dynamics in the Pleistocene of *B. belcheri* and *B. floridae* populations. The PSMC results showed that the inferred ancestral *N*_e_ of the two Chinese *B. belcheri* populations was different before the first glaciation (PG, 0.9−1.2Mya, mutation rate = 3×10^−9^) of the Pleistocene (Figure 5a), indicating that the two populations most probably started to evolve in isolation in their respective seas after the first glaciation of the Pleistocene. When applying the higher mutation rate of 9 × 10^−9^, the divergence time of the Zhanjiang and Xiamen *B. belcheri* populations should be postponed to the third glaciation of the Pleistocene (Figure 5c). The different habitats of Chinese *B. belcheri* probably stemmed from glacial flows after the first or third glaciation of the Pleistocene, which caused some *B. belcheri* to leave their original habitats to migrate to warmer seas to survive. Interestingly, as shown in Figure 6, the PSMC results of three *B. floridae* individuals showed that the laboratory‐raised *B. floridae* from China shared almost the same demographic history with the two wild *B. floridae* from Tampa Bay, USA, suggesting that the three individuals (BF_CN1, BF_TP1, and BF_TP2) belonged to the same population.

Many previous studies have reported dramatic population contractions during the LGP, followed by a population bottleneck during the last glacial maximum (LGM, 20–26.5 kya) (Clark et al., 2009). For example, in giant pandas, a population bottleneck occurred during the LGM, when substantial alpine glaciations would likely have resulted in the extensive loss of panda habitats (Zhao et al., 2013); in eagle, ostrich, and many other avian populations, a clear and drastic reduction in *N_e_* occurred at the beginning of or during the LGP, when their survival habitats contracted or disappeared due to the glacial climatic fluctuation (Nadachowska‐Brzyska et al., 2015). Moreover, rhinoceros, bear, and many other species also suffered from dramatic population reductions at the beginning of the LGP and reached a population bottleneck during the LGM (Miller et al., 2012). In this study, we also detected population reductions in the *Branchiostoma* populations at the beginning of the LGP and found a population bottleneck during the LGM (Figures 5 and 6). At the beginning of the LGP, the PSMC curves of the seven *B. belcheri* individuals from China all showed a severe population reduction, while the three *B. floridae* individuals only experienced a minor population reduction during this period (mutation rate = 3 × 10^−9^). This may be due to the fact that the *B. floridae* population from America was subject to a very long‐term population reduction before the LGP, and thus, the population size was very small during the LGP. Moreover, the time points of population bottlenecks between *B. belcheri* and *B. floridae* were slightly different, which may be due to the different levels of glaciation in Eastern China and North America. However, it should be noted that the resolution of the PSMC analysis was poor in recent times (including the LGP), especially since 20 kya (Li & Durbin, 2011; Nadachowska‐Brzyska et al., 2015). We performed 100 bootstrap replicates to assess the robustness of our PSMC results during the Pleistocene. As shown in Figures 5 and 6, the bootstrapping PSMC curves confirmed the accuracy of our methods in most of the Pleistocene. However, in the more recent past (<20 kya), there is considerable variation in effective population size estimates among bootstrap replicates for the most recent time intervals, confirming the limitations of PSMC analysis to infer demography in the more recent past (Li & Durbin, 2011).

Amphioxus has always been considered as a promising model animal to study the evolutionary and developmental mechanisms of vertebrates because of its unique phylogenetic position and simple body plan. Amphioxus species are distributed throughout the world along tropical and temperate coasts in habitats with a suitable temperature and salinity and a sandy seabed. Before the 1960s, along parts of the coast of China, amphioxus species were so numerous that they constituted the basis of a fishing industry (Chin, 1941; Light, 1923). Although edible, amphioxus species have never been sufficiently abundant to constitute a significant source of food to humans or to be an important part of the food chain in nature. With the severe industrial pollution and the construction of seawalls, the habitats of amphioxus species have been destroyed. At present, amphioxus species have been listed in the registry of “State Second‐Class Protected Animals” in China and “Endangered Animals of Japanese Marine and Fresh Water Organisms” in Japan. We are currently in a warm interglacial period of the Holocene, which is suitable for the breeding of amphioxus species. Therefore, we should provide sanctuaries for the amphioxus species rather than destroying their habitats for human development.

## CONFLICT OF INTEREST

The authors report no conflict of interest.

## AUTHOR CONTRIBUTION

**Changwei Bi:** Methodology (equal); Project administration (lead); Software (lead); Visualization (lead); Writing‐original draft (lead); Writing‐review & editing (lead). **Na Lu:** Methodology (equal); Software (equal); Visualization (equal). **Zhen Huang:** Data curation (equal); Funding acquisition (equal); Resources (equal). **J.‐Y. Chen:** Conceptualization (equal); Data curation (equal); Resources (equal). **Chunpeng He:** Data curation (equal); Funding acquisition (lead); Investigation (equal); Methodology (equal); Project administration (lead); Resources (lead); Supervision (equal); Writing‐review & editing (equal). **Zuhong Lu:** Conceptualization (lead); Funding acquisition (lead); Methodology (equal); Project administration (lead); Resources (equal); Supervision (lead); Validation (equal); Writing‐review & editing (equal).

## Supporting information

Supplementary Material

## Data Availability

All sequencing data in this study have been submitted to the NCBI Short Read Archive (SRA) under the BioProject accession: PRJNA573992 (SRA accessions: SRR10176999, SRR10177000 and SRR10177001). The other sequencing data used in this study were downloaded from NCBI SRA database (SRA accessions: SRR1174914, SRR1174915, SRR1174916, SRR1174917, SRR1174918, SRR6162896, and SRR6162897). The reference genomes from *B. belcheri* v. 18h27 (GenBank assembly accession: GCF_001625305.1) and *B. floridae* v.2.0 (GenBank assembly accession: GCF_000003815.1) were downloaded from NCBI genome database (https://www.ncbi.nlm.nih.gov/genome/browse). All scripts used in this study are available at https://github.com/bichangwei/Branchiostoma_SNP. The authors declare that all other data supporting the findings of this study are available within the article and its electronic supplementary material files.
