# Supplementary material for: Whole‐genome resequencing reveals the pleistocene temporal dynamics of Branchiostoma belcheri and Branchiostoma floridae populations
Source: Ecol Evol. 2020 Jul 20;10(15):8210–24. doi: 10.1002/ece3.6527 (PMC7417228; doi:10.1002/ece3.6527)
Supplement: Supplementary file 1 — Supplementary Material [file ECE3-10-8210-s001.docx]

**Whole-Genome Resequencing Reveals the** **Pleistocene Temporal Dynamics of *Branchiostoma* *belcheri* and *Branchiostoma floridae* Populations**

Changwei Bi^1^, Na Lu^1^, Zhen Huang^2,3^, J.-Y. Chen^4^, Chunpeng He^1, *^, Zuhong Lu^1, *^

**Supporting Information:**

**Figure S1. Distribution of variants in different genomic regions of *Branchiostoma*.** The variants detected in intronic, intergenic, up-downstream, UTR, and exonic regions are shown in purple, green, blue, baby blue and red, respectively.
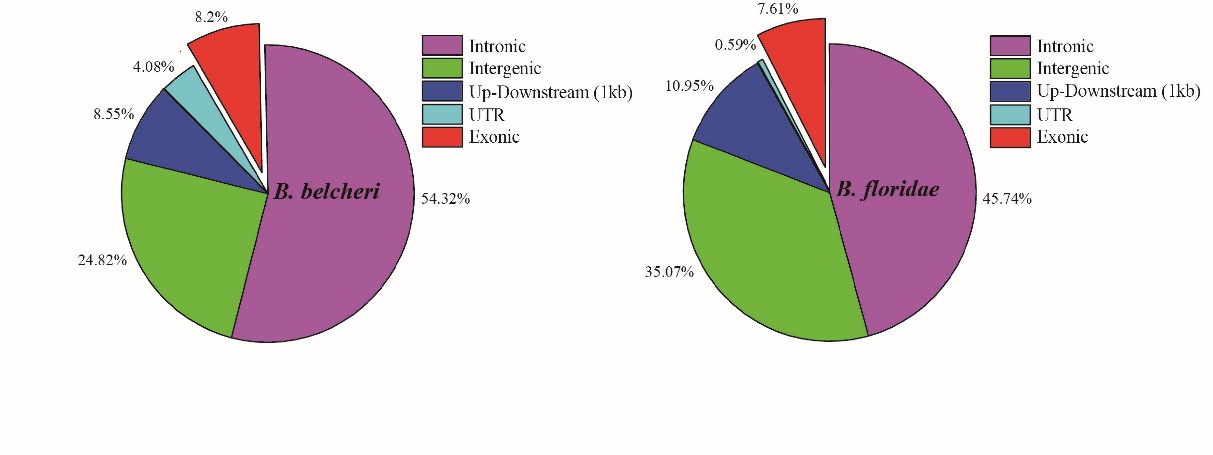


**Figure S2. Distribution of exonic indels lengths in seven selected *Branchiostoma* genomes.** The horizontal axis represents the length of indels, and the vertical axis represents the ratio of the number of indels of the length.


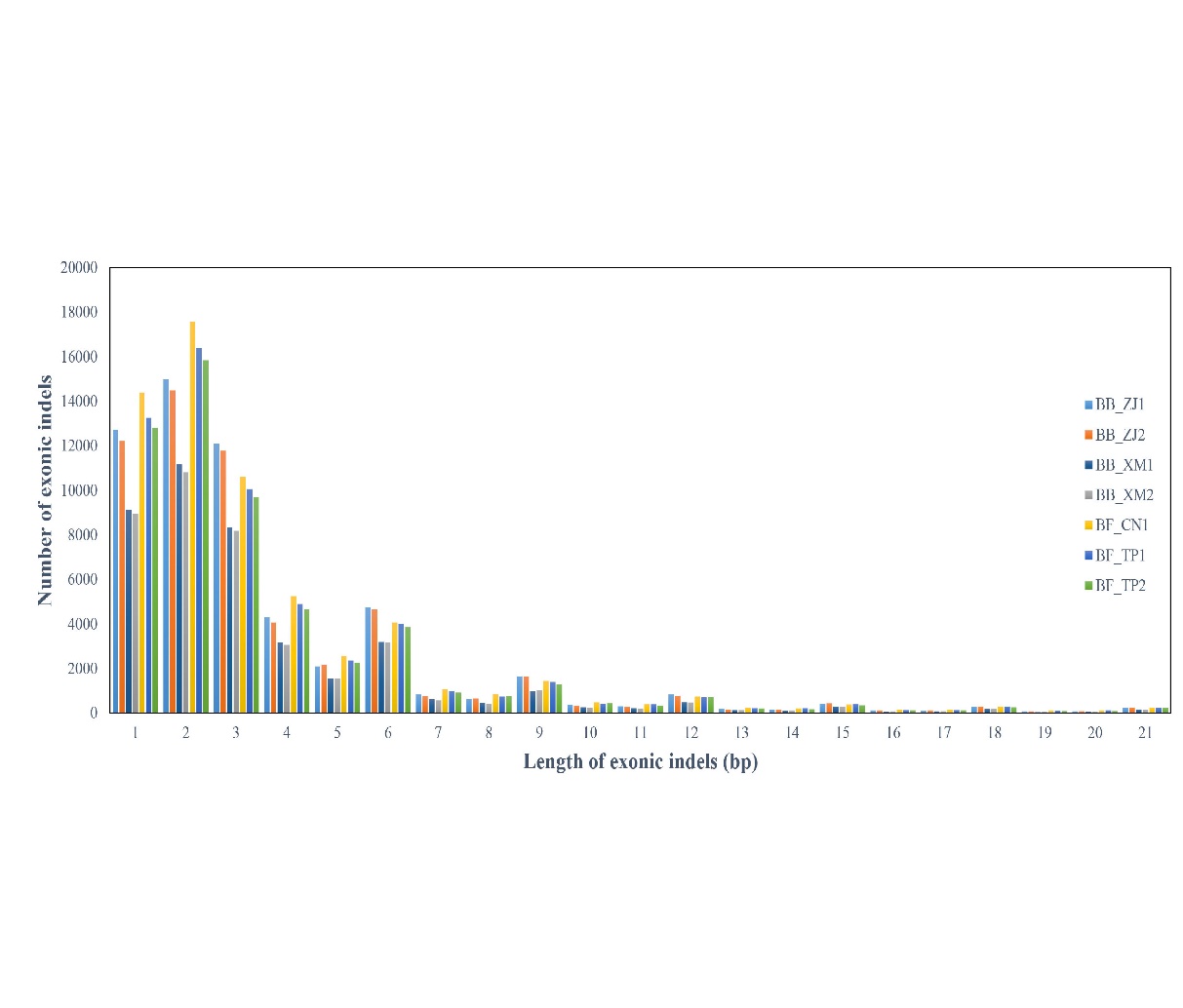


**Figure S3. Temporal dynamics of effective population size for all ten *B. belcheri* individuals.**

**
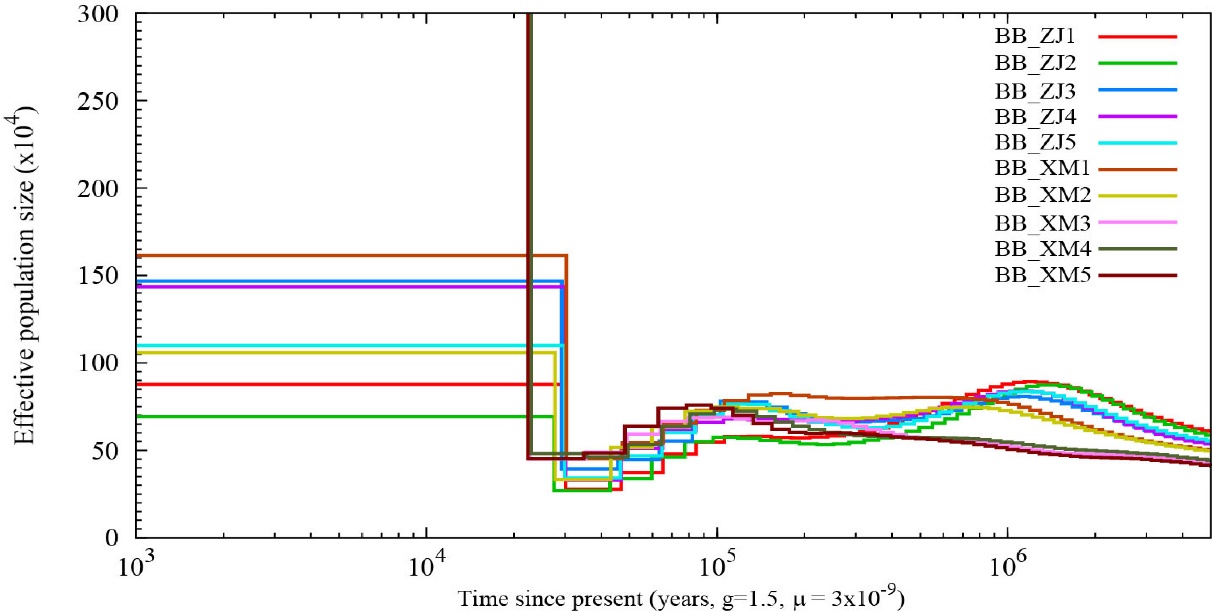
**

**Figure S4. Temporal dynamics of effective population size for all ten *B. floridae* individuals.
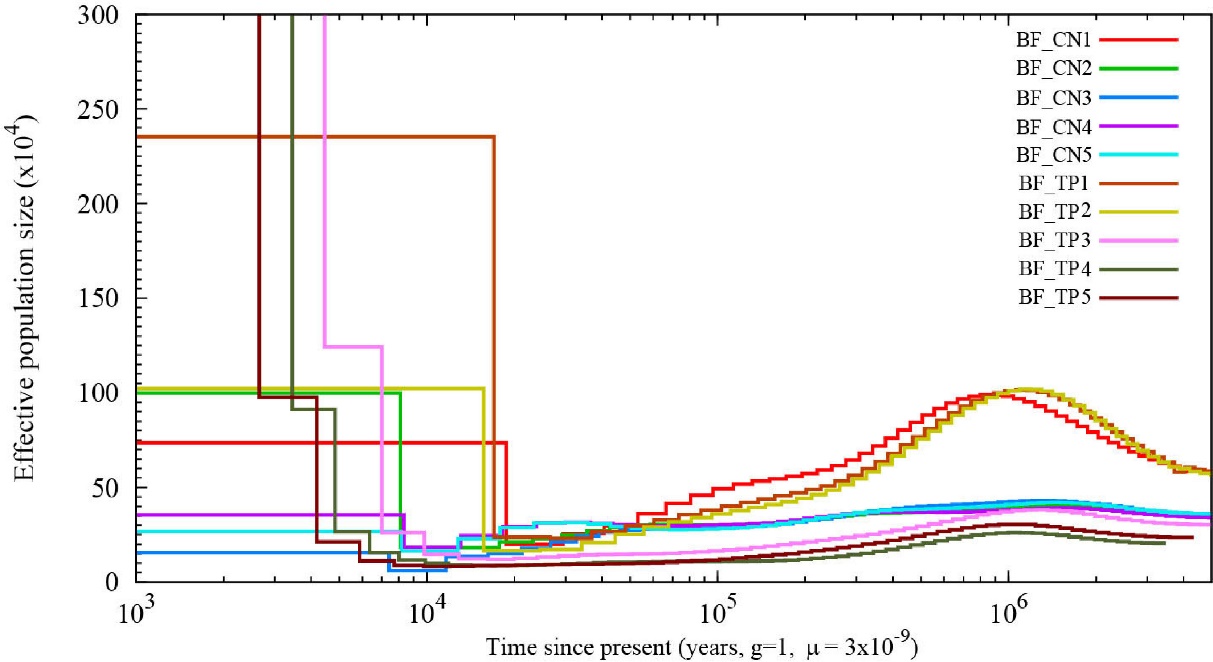
**

**Figure S5. The influence of recombination rates (r) on PSMC results.** The colors of PSMC curves indicate different recombination rates.

**
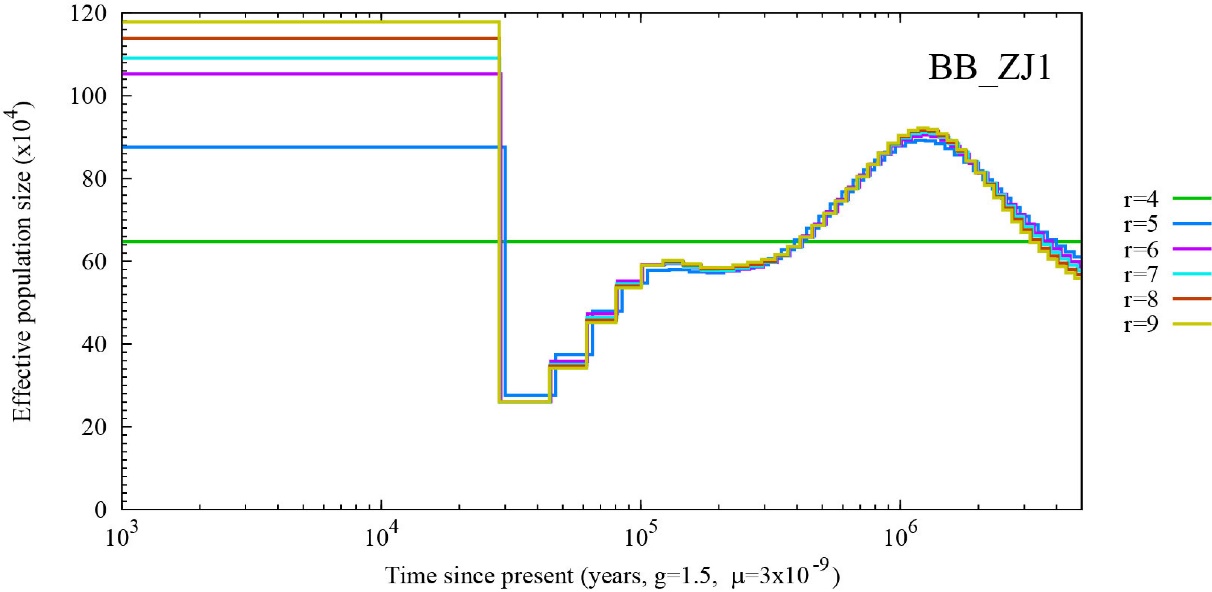
**

**Figure S6. The influence of generation time (g) on PSMC results.** Using the fixed mutation rate of 3×10^-9^, the PSMC results for BB_ZJ1 and BB_ZJ2 generated from the generation time of 1.5 and 3.

**
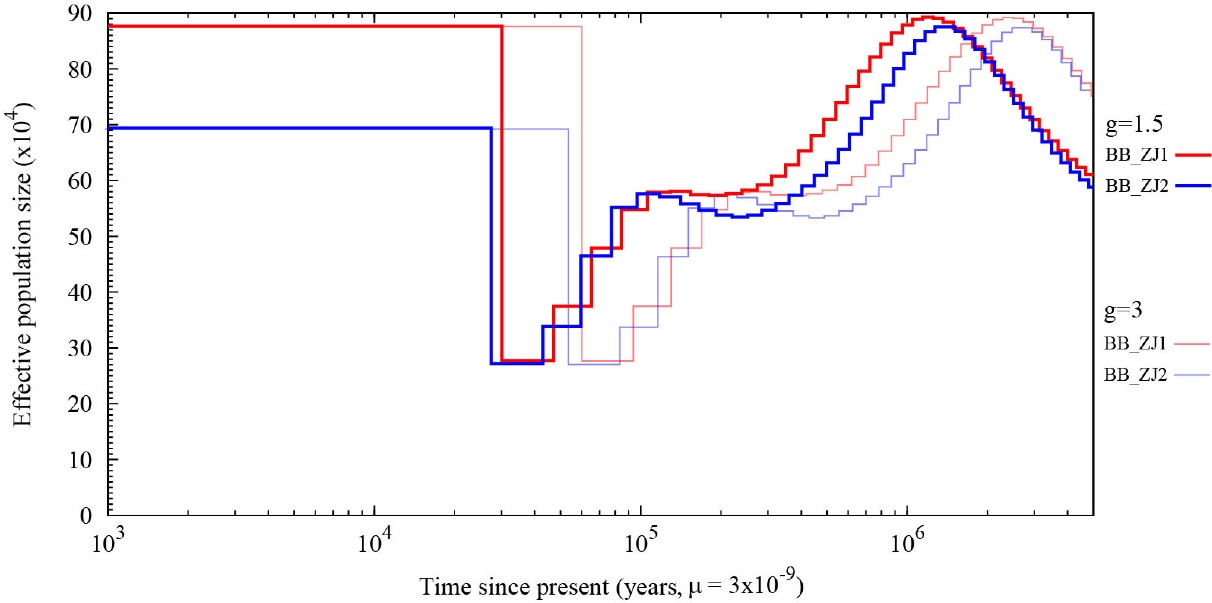
**

**Figure S7. The influence of per generation mutation rate (μ) on PSMC results.** Using the fixed generation time of 1.5, the PSMC results for BB_ZJ1 and BB_ZJ2 generated from the mutation rates of 3×10^-9^ and 6×10^-9^.


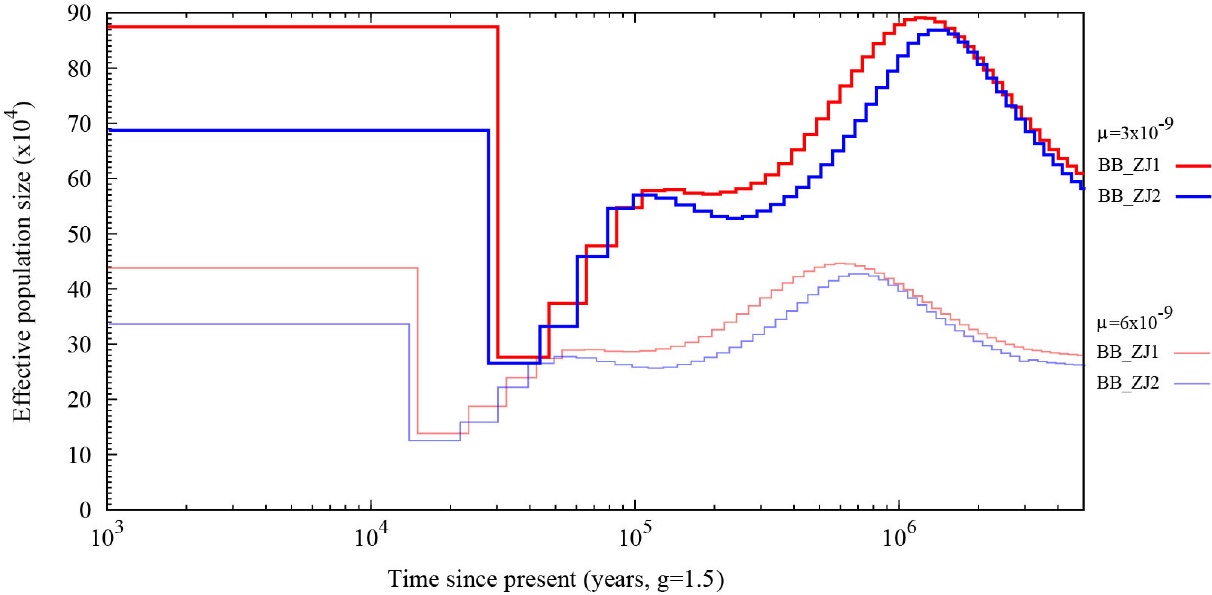


**Table S1. Description of variants before and after stringent filtrations.**

| Sample | Original Variations | | | Filtered Variations | | | Filtration rate |
| --- | --- | --- | --- | --- | --- | --- | --- |
|  | SNV | Indel | Total | SNV | Indel | Total |  |
| BB_ZJ1 | 13,256,531 | 4,001,163 | 17,257,694 | 10,982,867 | 3,747,782 | 14,730,649 | 14.64% |
| BB_ZJ2 | 13,005,102 | 3,913,750 | 16,918,852 | 10,701,059 | 3,667,946 | 14,369,005 | 15.07% |
| BB_ZJ3 | 12,979,489 | 3,788,418 | 16,767,907 | 10,923,216 | 3,608,456 | 14,531,672 | 13.34% |
| BB_ZJ4 | 12,962,149 | 3,787,132 | 16,749,281 | 10,923,224 | 3,605,584 | 14,528,808 | 13.26% |
| BB_ZJ5 | 12,985,448 | 3,797,647 | 16,783,095 | 10,874,521 | 3,624,716 | 14,499,237 | 13.61% |
| BB_XM1 | 12,916,608 | 3,762,736 | 16,679,344 | 10,567,400 | 3,453,744 | 14,021,144 | 15.94% |
| BB_XM2 | 12,771,588 | 3,716,013 | 16,487,601 | 10,346,899 | 3,412,090 | 13,758,989 | 16.55% |
| BB_XM3 | 12,360,709 | 3,546,431 | 15,907,140 | 11,132,435 | 3,399,371 | 14,531,806 | 8.65% |
| BB_XM4 | 12,440,724 | 3,574,036 | 16,014,760 | 11,206,853 | 3,425,448 | 14,632,301 | 8.63% |
| BB_XM5 | 12,343,327 | 3,535,739 | 15,879,066 | 11,125,811 | 3,393,591 | 14,519,402 | 8.56% |
| BF_CN1 | 15,123,133 | 3,928,727 | 19,051,860 | 12,458,223 | 3,582,871 | 16,041,094 | 15.80% |
| BF_CN2 | 13,236,608 | 3,202,303 | 16,438,911 | 11,669,565 | 3,045,151 | 14,714,716 | 10.49% |
| BF_CN3 | 13,099,891 | 3,175,342 | 16,275,233 | 11,472,061 | 3,006,838 | 14,478,899 | 11.04% |
| BF_CN4 | 13,154,112 | 3,154,416 | 16,308,528 | 11,572,173 | 2,988,540 | 14,560,713 | 10.72% |
| BF_CN5 | 13,356,911 | 3,251,608 | 16,608,519 | 11,772,796 | 3,093,965 | 14,866,761 | 10.49% |
| BF_TP1 | 14,575,170 | 3,761,195 | 18,336,365 | 12,695,025 | 3,477,219 | 16,172,244 | 11.80% |
| BF_TP2 | 14,163,886 | 3,611,519 | 17,775,405 | 12,270,537 | 3,340,406 | 15,610,943 | 12.18% |
| BF_TP3 | 12,589,376 | 3,014,416 | 15,603,792 | 11,091,968 | 2,888,518 | 13,980,486 | 10.40% |
| BF_TP4 | 11,517,388 | 2,643,322 | 14,160,710 | 10,107,576 | 2,536,289 | 12,643,865 | 10.71% |
| BF_TP5 | 11,902,417 | 2,804,339 | 14,706,756 | 10,441,452 | 2,686,818 | 13,128,270 | 10.73% |

**Table S2. The functional annotation statistics of SNVs in *Branchiostoma*.**

| Variant type | | *B. belcheri* | | | | | | | | | | |
| --- | --- | --- | --- | --- | --- | --- | --- | --- | --- | --- | --- | --- |
|  |  | BB_ZJ1 | BB_ZJ2 | BB_ZJ3 | BB_ZJ4 | BB_ZJ5 | BB_XM1 | BB_XM2 | BB_XM3 | BB_XM4 | BB_XM5 |  |
| Total SNV | | 10,982,867 | 10,701,059 | 10,923,216 | 10,923,224 | 10,874,521 | 10,567,400 | 10,346,899 | 11,132,435 | 11,206,853 | 11,125,811 |  |
| Ti | | 6,237,694 | 6,080,525 | 6,200,876 | 6,198,136 | 6,172,295 | 5,966,013 | 5,842,952 | 6,314,846 | 6,357,310 | 6,311,991 |  |
| Tv | | 4,745,173 | 4,620,534 | 4,722,340 | 4,725,088 | 4,702,226 | 4,601,387 | 4,503,947 | 4,817,589 | 4,849,543 | 4,813,820 |  |
| Ti/Tv | | 1.3145 | 1.3160 | 1.3131 | 1.3118 | 1.3126 | 1.2966 | 1.2973 | 1.3108 | 1.3109 | 1.3112 |  |
| Intronic | | 5,711,882 | 5,563,478 | 5,689,896 | 5,699,506 | 5,663,449 | 5,665,353 | 5,551,117 | 5,764,236 | 5,800,884 | 5,753,046 |  |
| Intergenic | | 2,714,380 | 2,639,182 | 2,703,405 | 2,704,464 | 2,688,115 | 2,712,732 | 2,639,646 | 2,784,095 | 2,806,492 | 2,787,353 |  |
| Upstream(1kb) | | 413,764 | 403,911 | 418,317 | 417,790 | 417,017 | 404,079 | 393,570 | 421,767 | 427,219 | 422,617 |  |
| Downstream(1kb) | | 423,206 | 415,315 | 425,426 | 424,644 | 422,846 | 422,328 | 415,349 | 429,491 | 430,437 | 427,603 |  |
| Upstream; Downstream | | 66,257 | 65,783 | 66,942 | 67,256 | 67,255 | 64,693 | 63,723 | 67,095 | 66,719 | 66,127 |  |
| Splicing | | 1,272 | 1,249 | 1,336 | 1,262 | 1,256 | 1,187 | 1,196 | 1,372 | 1,381 | 1,318 |  |
| UTR | | 443,684 | 433,159 | 451,977 | 449,480 | 449,473 | 417,603 | 408,942 | 444,753 | 447,156 | 443931 |  |
| Exonic | Total | 1,208,422 | 1,178,982 | 1,165,917 | 1,158,822 | 1,165,110 | 879,425 | 873,356 | 1,219,626 | 1,226,565 | 1,223,816 |  |
|  | Non-synonymous | 354,978 | 344,590 | 336,849 | 335,619 | 337,818 | 254,134 | 250,606 | 361,172 | 364,613 | 361,964 |  |
|  | Synonymous | 850,689 | 831,797 | 826,597 | 820,622 | 824,741 | 623,179 | 620,623 | 855,602 | 859,124 | 858,989 |  |
|  | Stop gained | 2,351 | 2,222 | 2,087 | 2,195 | 2,169 | 1,757 | 1,811 | 2,444 | 2,426 | 2,455 |  |
|  | Stop loss | 404 | 373 | 384 | 386 | 382 | 355 | 316 | 408 | 402 | 408 |  |
|  | Ti/Tv | 1.8067 | 1.8065 | 1.8150 | 1.8088 | 1.8124 | 1.8009 | 1.8092 | 1.7943 | 1.7868 | 1.7973 |  |
|  |  |  |  |  |  |  |  |  |  |  |  |  |
| Variant type | | *B. floridae* | | | | | | | | | | |
|  |  | BF_CN1 | BF_CN2 | BF_CN3 | BF_CN4 | BF_CN5 | BF_TP1 | BF_TP2 | BF_TP3 | BF_TP4 | BF_TP5 |  |
| Total SNV | | 12,458,223 | 11,669,565 | 11,472,061 | 11,572,173 | 11,772,796 | 12,695,025 | 12,270,537 | 11,091,968 | 10,107,576 | 10,441,452 |  |
| Ti | | 7,074,220 | 6,625,767 | 6,510,690 | 6,570,778 | 6,685,609 | 7,210,371 | 6,974,650 | 6,299,381 | 5,742,739 | 5,929,330 |  |
| Tv | | 5,384,003 | 5,043,798 | 4,961,371 | 5,001,395 | 5,087,187 | 5,484,654 | 5,295,887 | 4,792,587 | 4,364,837 | 4,512,122 |  |
| Ti/Tv | | 1.3139 | 1.3137 | 1.3123 | 1.3138 | 1.3142 | 1.3146 | 1.3170 | 1.3144 | 1.3157 | 1.3141 |  |
| Intronic | | 5,533,618 | 5,185,477 | 5,097,505 | 5,148,032 | 5,229,919 | 5,628,661 | 5,440,065 | 4,932,594 | 4,489,123 | 4,650,335 |  |
| Intergenic | | 4,366,837 | 4,078,952 | 4,010,706 | 4,046,973 | 4,122,046 | 4,469,191 | 4,306,858 | 3,872,265 | 3,527,878 | 3,637,496 |  |
| Upstream(1kb) | | 641,754 | 601,252 | 588,873 | 594,380 | 603,720 | 649,797 | 630,806 | 572,606 | 519,262 | 539,234 |  |
| Downstream(1kb) | | 651,868 | 611,371 | 601,174 | 601,050 | 614,627 | 659,519 | 639,168 | 585,223 | 529,318 | 550,437 |  |
| Upstream; Downstream | | 50,865 | 46,587 | 46,909 | 46,624 | 47,891 | 51,057 | 48,948 | 44,657 | 40,841 | 41,650 |  |
| Splicing | | 2,603 | 2,558 | 2,455 | 2,460 | 2,489 | 2,682 | 2,675 | 2,384 | 2,147 | 2,258 |  |
| UTR | | 72,542 | 67,893 | 66,428 | 67,169 | 68,748 | 73,852 | 71,247 | 66,603 | 60,712 | 63,206 |  |
| Exonic | Total | 1,138,136 | 1,075,475 | 1,058,011 | 1,065,485 | 1,083,356 | 1,160,266 | 1,130,770 | 1,015,636 | 938,295 | 956,836 |  |
|  | Non-synonymous | 381,380 | 360,801 | 356,547 | 357,827 | 363,106 | 393,285 | 382,671 | 338,865 | 313,649 | 319,199 |  |
|  | Synonymous | 752,367 | 710,549 | 697,279 | 703,610 | 716,141 | 762,542 | 743,738 | 672,889 | 621,081 | 634,056 |  |
|  | Stop gained | 3,737 | 3,556 | 3,591 | 3,441 | 3,470 | 3,786 | 3,724 | 3,309 | 2,994 | 3,056 |  |
|  | Stop loss | 652 | 569 | 594 | 607 | 639 | 653 | 637 | 573 | 571 | 525 |  |
|  | Ti/Tv | 1.7995 | 1.7897 | 1.7906 | 1.7925 | 1.7930 | 1.8017 | 1.7957 | 1.8068 | 1.8013 | 1.8037 |  |

**Table S3. The functional annotation statistics of indels in *Branchiostoma*.**

| Variant type | | *B. belcheri* | | | | | | | | | |
| --- | --- | --- | --- | --- | --- | --- | --- | --- | --- | --- | --- |
|  |  | BB_ZJ1 | BB_ZJ2 | BB_ZJ3 | BB_ZJ4 | BB_ZJ5 | BB_XM1 | BB_XM2 | BB_XM3 | BB_XM4 | BB_XM5 |
| Total | | 3,747,782 | 3,667,946 | 3,608,456 | 3,605,584 | 3,624,716 | 3,453,744 | 3,412,090 | 3,399,371 | 3,425,448 | 3,393,591 |
| Insertion | | 1,875,004 | 1,835,718 | 1,781,292 | 1,779,643 | 1,790,614 | 1,728,791 | 1,707,614 | 1,687,239 | 1,698,781 | 1,683,332 |
| Deletion | | 1,872,778 | 1,832,228 | 1,827,164 | 1,825,941 | 1,834,102 | 1,724,953 | 1,704,476 | 1,712,132 | 1,726,667 | 1,710,259 |
| Intergenic | | 915,547 | 895,700 | 881,458 | 880,643 | 889,615 | 841,000 | 828,458 | 818,651 | 824,671 | 818,832 |
| Intronic | | 2,268,775 | 2,221,201 | 2,181,567 | 2,180,321 | 2,186,757 | 2,105,669 | 2,083,558 | 2,064,447 | 2,078,955 | 2,056,483 |
| Upstream (1kb) | | 151,843 | 148,277 | 147,073 | 146,808 | 148,101 | 139,253 | 138,199 | 138,617 | 140,378 | 139,679 |
| Downstream (1kb) | | 167,697 | 164,111 | 161,806 | 161,691 | 162,859 | 155,399 | 153,806 | 152,518 | 153,088 | 152,726 |
| Upstream; Downstream | | 27,625 | 27,455 | 26,794 | 26,775 | 27,116 | 25,878 | 25,283 | 25,216 | 24,951 | 25,170 |
| Splicing | | 3,474 | 3,356 | 3,413 | 3,418 | 3,407 | 3,164 | 3,151 | 3,189 | 3,223 | 3,234 |
| UTR | | 155,191 | 151,902 | 152,807 | 152,719 | 152,903 | 142,241 | 139,526 | 145,337 | 147,551 | 145,617 |
| Exonic | Total | 57,630 | 55,944 | 53,538 | 53,209 | 53,958 | 41,140 | 40,109 | 51,396 | 52,631 | 51,850 |
|  | Non-frameshift | 20,242 | 19,847 | 19,085 | 18,777 | 18,977 | 13,588 | 13,373 | 18,308 | 18,716 | 18,405 |
|  | Frameshift | 36,258 | 34,975 | 33,396 | 33,341 | 33,939 | 26,636 | 25,905 | 32,090 | 32,904 | 32,467 |
|  | Stop gain | 1,039 | 1,042 | 971 | 998 | 947 | 846 | 761 | 918 | 924 | 896 |
|  | Stop loss | 91 | 80 | 86 | 93 | 95 | 70 | 70 | 80 | 87 | 82 |
|  |  |  |  |  |  |  |  |  |  |  |  |
| Variant type | | *B. floridae* | | | | | | | | | |
|  |  | BF_CN1 | BF_CN2 | BF_CN3 | BF_CN4 | BF_CN5 | BF_TP1 | BF_TP2 | BF_TP3 | BF_TP4 | BF_TP5 |
| Total | | 3,582,871 | 3,045,151 | 3,006,838 | 2,988,540 | 3,093,965 | 3,477,219 | 3,340,406 | 2,888,518 | 2,536,289 | 2,686,818 |
| Insertion | | 1,788,263 | 1,512,063 | 1,493,238 | 1,480,731 | 1,535,304 | 1,752,202 | 1,684,300 | 1,435,056 | 1,260,733 | 1,337,167 |
| Deletion | | 1,794,608 | 1,533,088 | 1,513,600 | 1,507,809 | 1,558,661 | 1,725,017 | 1,656,106 | 1,453,462 | 1,275,556 | 1,349,651 |
| Intergenic | | 1,267,442 | 1,075,104 | 1,062,942 | 1,057,717 | 1,094,119 | 1,228,870 | 1,182,980 | 1,017,779 | 894,664 | 945,408 |
| Intronic | | 1,817,435 | 1,542,121 | 1,521,464 | 1,513,642 | 1,566,575 | 1,767,339 | 1,693,012 | 1,464,307 | 1,284,972 | 1,363,273 |
| Upstream (1kb) | | 195,287 | 168,085 | 165,937 | 164,096 | 169,347 | 188,694 | 181,738 | 159,097 | 139,395 | 148,375 |
| Downstream (1kb) | | 201,972 | 174,392 | 171,493 | 169,371 | 177,170 | 195,834 | 190,191 | 168,259 | 146,450 | 156,259 |
| Upstream; Downstream | | 15,693 | 13,075 | 13,339 | 12,929 | 13,629 | 14,944 | 14,114 | 12,362 | 11,049 | 11,527 |
| Splicing | | 2,576 | 2,274 | 2,182 | 2,247 | 2,292 | 2,385 | 2,351 | 2,045 | 1,841 | 1,940 |
| UTR | | 20,650 | 18,920 | 18,334 | 18,542 | 19,201 | 21,035 | 20,229 | 18,858 | 16,806 | 17,872 |
| Exonic | Total | 61,816 | 51,180 | 51,147 | 49,996 | 51,632 | 58,118 | 55,791 | 45,811 | 41,112 | 42,164 |
|  | Non-frameshift | 17,688 | 15,013 | 14,932 | 14,775 | 15,040 | 17,163 | 16,357 | 13,740 | 12,478 | 12,757 |
|  | Frameshift | 42,513 | 34,813 | 34,908 | 33,915 | 35,255 | 39,438 | 38,005 | 30,931 | 27,621 | 28,362 |
|  | Stop gain | 1,477 | 1,232 | 1,191 | 1,203 | 1,226 | 1,380 | 1,310 | 1,032 | 916 | 943 |
|  | Stop loss | 138 | 122 | 116 | 103 | 111 | 137 | 119 | 108 | 97 | 102 |
